# Effect of Band Bending in Photoactive MOF-Based Heterojunctions

**DOI:** 10.1021/acsami.2c00335

**Published:** 2022-04-20

**Authors:** Giulia E. M. Schukraft, Benjamin Moss, Andreas G. Kafizas, Camille Petit

**Affiliations:** † Barrer Centre, Department of Chemical Engineering, South Kensington Campus, Imperial College London, London SW7 2AZ, U.K.; ‡ Department of Materials, South Kensington Campus, Imperial College London, London SW7 2AZ, U.K.; § Department of Chemistry, Molecular Science Research Hub, White City Campus, Imperial College London, London W12 0BZ, U.K.; ∥ The Grantham Institute, Imperial College London, London SW7 2AZ, U.K.

**Keywords:** photocatalysis, CO_2_ reduction, heterojunction, metal−organic frameworks, graphitic carbon nitride, band bending

## Abstract

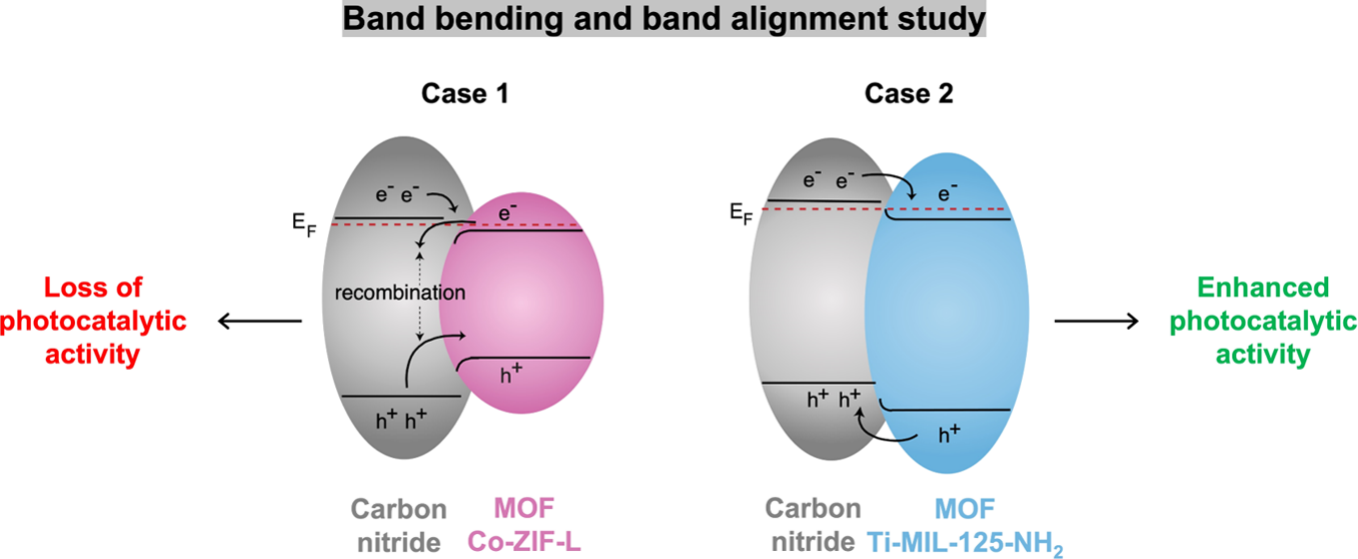

Semiconductor/metal–organic framework (MOF) heterojunctions have demonstrated promising performance for the photoconversion of CO_2_ into value-added chemicals. To further improve performance, we must understand better the factors which govern charge transfer across the heterojunction interface. However, the effects of interfacial electric fields, which can drive or hinder electron flow, are not commonly investigated in MOF-based heterojunctions. In this study, we highlight the importance of interfacial band bending using two carbon nitride/MOF heterojunctions with either Co-ZIF-L or Ti-MIL-125-NH_2_. Direct measurement of the electronic structures using X-ray photoelectron spectroscopy (XPS), work function, valence band, and band gap measurements led to the construction of a simple band model at the heterojunction interface. This model, based on the heterojunction components and band bending, enabled us to rationalize the photocatalytic enhancements and losses observed in MOF-based heterojunctions. Using the insight gained from a promising band bending diagram, we developed a Type II carbon nitride/MOF heterojunction with a 2-fold enhanced CO_2_ photoreduction activity compared to the physical mixture.

## Introduction

Identifying ways to efficiently harness renewable energy sources is a key driver in various research fields.^1^ Artificial photosynthesis, i.e., the photocatalytic conversion of water or CO_2_ into H_2_ or C_1+_ fuels, respectively, remains at the lower end of the technological readiness scale for renewable energy sources. The simplicity of its implementation and the remaining unknowns around photocatalyst design optimization, efficiency improvement, and large-scale deployment have fostered intense research in this field.^2,3^ In the context of CO_2_ conversion into value-added chemicals, the design of efficient photocatalysts remains a priority.^4−7^ Several semiconductors (e.g., TiO_2_, ZnO, CdS, WO_3_, and graphitic carbon nitride (g-C_3_N_4_)) have been extensively studied for photocatalytic CO_2_ conversion.^8−10^ Currently, however, challenges such as fast electron–hole recombination and poor utilization of the solar spectrum limit their efficiency.^9,11,12^

The formation of heterojunctions—i.e., the formation of an intimate contact between two distinct materials—may help address some of these shortcomings. Heterojunctions provide an interface across which carriers may become separated and, if materials with complementary band gaps are chosen, increase the proportion of the solar spectrum which can be harvested.^13^ Compared to conventional semiconductor heterojunctions, semiconductor/metal–organic framework (MOF) heterojunctions provide unique advantages, which have been explored in several studies.^14,15^ The high tunability of MOFs enables tailored structural, optoelectronic, and surface properties beyond those of many inorganic materials. In theory, one can select a MOF to display desirable physicochemical properties (e.g., CO_2_ affinity, pore sizes, surface area, light absorption) and build a MOF-based heterojunction with enhanced activity compared to its individual components.^16−19^ To date, numerous semiconductor/MOFs heterojunctions have been developed and investigated for use as catalysts for CO_2_ photoconversion into value-added chemicals.^6,16,18^

Morphological, structural, and chemical features of the heterojunction interface can influence the electron flow across the interface.^16,20−22^ Controlling and understanding this interface thereby support the design of efficient heterojunctions. For instance, the band bending, caused by an offset in Fermi levels between two heterojunction components, can affect electron/hole flow across the heterojunction under illumination. However, until now, this concept has been rarely studied in MOF-based heterojunctions.^23,24^

In this paper, we show how X-ray photoelectron spectroscopy (XPS), work function, valence band, and optical band gap measurements can be used to create a simple band model of the electronic structure of MOF-based heterojunction interfaces. Taking into account band bending and band alignment, the models enable us to rationalize the photocatalytic enhancements and losses in MOF-based heterojunctions based on their individual components. To illustrate our point, we built two types of heterojunctions, both using a graphitic carbon nitride as the semiconductor representative. One heterojunction involves Co-ZIF-L as the MOF, and the other involves Ti-MIL-125-NH_2_. We chose Co-ZIF-L and Ti-MIL-125-NH_2_ owing to their 2D morphology. The 2D–2D contact between the MOF and CNNS should favor a high surface contact and promote charge separation. In addition, both MOFs exhibit visible light absorption and an ability to catalyze CO_2_ photoreduction.^13,20,25^ Through materials characterization and CO_2_ photoreduction testing, we show that the effects of band bending and band alignment enable a better description of the charge transfer process occurring at a MOF-based heterojunction interface. Our findings helped us develop a Ti-MOF-based heterojunction, where we measured a ∼2-fold improvement in photocatalytic activity for the heterojunction compared to the physical mixture.

## Experimental Section

### Material Synthesis

All reagents used in this study were of analytical grade and used without further purification. Co(NO_3_)_2_·6H_2_O (98%), 2-methylimidazole (2-Hmin, 99%), titanium isopropoxide (97%), melamine (99%), and P25 TiO_2_ were purchased from Sigma-Aldrich.

#### Graphitic Carbon Nitride Nanosheets (CNNS-1 and CNNS-2)

The synthesis approach was adapted from ref (26). Bulk graphitic carbon nitride (g-C_3_N_4_) was synthesized from heating melamine in a muffle furnace to 560 °C at a 5 °C/min ramp under static air and maintaining the temperature for 4 h. CNNS were obtained by liquid exfoliation of the prepared g-C_3_N_4_. Briefly, 500 mg of bulk g-C_3_N_4_ was ultrasonicated (Elma, Elmasonic P H 30) into 150 mL of a 75:25 water/ethanol mixture. Two batches of CNNS were prepared and are referred to as CNNS-1 and CNNS-2. For CNNS-1, after 8 h of sonication, the graphic nanosheets in the supernatant were collected by centrifugation at 1000 rpm for 4 min. For CNNS-2, after 5 h of sonication, the graphic nanosheets in the supernatant were collected by centrifugation at 1000 rpm for 2 min. The final products were dried at 110 °C overnight in air.

#### Co-ZIF-L

The synthesis approach was adapted from ref (27). Typically, 0.625 g of Co(NO_3_)_2_.6H_2_O and 1.3 g of 2-Hmin were respectively dissolved in 40 mL of deionized water. The metal-containing solution was then rapidly added to the 2-Hmin solution under magnetic stirring. After 30 min of stirring, the reaction was left idle for 4 h. The final product was centrifuged, washed twice with ethanol, and dried at 110 °C overnight in air. Briefly, 0.5 g of the product was recovered.

#### Ti-MIL-125-NH_2_

The synthesis approach was adapted from ref (28). Briefly, 180.4 mg of 2-amino benzenedicarboxylic acid was mixed with 4.15 mL of DMF and 4.15 mL of methanol. Then, 0.148 mL of titanium isopropoxide was added dropwise under magnetic stirring. The solution was then sonicated for 5 min. The solution was transferred to a 25 mL autoclave and heated to 150 °C for 16 h. After natural cooling to room temperature, the obtained yellow product was washed with DMF and methanol and dried at 150 °C overnight under air. Briefly, 130 mg of the product was recovered.

#### CNNS-1/Co-ZIF-L Heterojunctions

A hydrothermal in situ synthesis was used to synthesize Co-ZIF-L in the presence of preformed CNNS to obtain CNNS/Co-ZIF-L heterojunctions. The desired quantity of CNNS-1 was ultrasonicated in the presence of 125 mg of Co(NO_3_)_2_ in 8 mL of deionized water for 30 min. Then, 260 mg of 2-Hmim was dissolved into 8 mL of deionized water. Once the sonication was complete, the Co(NO_3_)_2_ solution was added to the 2-Hmim solution under vigorous stirring. After 30 min of stirring, the reaction was left idle for 4 h. The solution was centrifuged, and the supernatant was removed. The final product was dried at 60 °C overnight in air. The obtained heterojunctions are referred to CNNS-1/ZIF-L-*X*, where *X* represents the weight percent of MOF in the sample, as determined by thermogravimetric analysis (TGA, Figure S1 and Table S1). *X* ranges from 8 to 96%.

#### CNNS-2/Ti-MIL-125-NH_2_ Heterojunction

A similar procedure to CNNS-1/Co-ZIF-L heterojunctions was used here. Typically, 432 mg of CNNS-2 was ultrasonicated in the presence of 180 mg of 2-aminoterephtalic acid in 4.15 mL of DMF and 4.15 mL of methanol for 30 min. Then, 0.148 mL of titanium isopropoxide was added dropwise under magnetic stirring. The solution was sonicated for 5 min. The solution was then transferred to a 25 mL autoclave and heated to 150 °C for 16 h. After cooling to room temperature, the obtained yellow product was washed with DMF and methanol and dried at 150 °C overnight. The obtained heterojunction is referred to as CNNS-2/MIL-25, where the number 25 represents the weight percent of MOF in the sample, as determined by TGA (Figure S2 and Table S2).

### Materials Characterization Methods

#### Chemical Properties

Thermal analyses were performed using a Netzsch TG209 F1 Libra thermogravimetric analyzer following the same procedure as reported before.^29^ Specifically, at least 10 mg of the sample was heated from room temperature to 900 °C at a rate of 10 °C min^–1^ under a N_2_ gas flow (flow rate 100 mL min^–1^). An isothermal step of 1 h was included at 120 °C to ensure the removal of adsorbates before heating was continued. Fourier transform infrared (FTIR) spectroscopy was performed using a PerkinElmer Spectrum 100 FTIR spectrometer equipped with an attenuated total reflectance (ATR) accessory following the same procedure as reported before.^29,30^ Specifically, 16 spectra were collected per sample to obtain an averaged spectrum over the frequency range of 450–4000 cm^–1^ with a resolution of 2 cm^–1^. X-ray photoelectron spectroscopy (XPS) measurements were carried out on a Thermo Scientific K-Alpha+ X-ray photoelectron spectrometer equipped with a MXR3 Al Kα monochromated X-ray source (hν = 1486.6 eV) following the same procedure as reported before.^29−31^ Specifically, the samples were mounted on the XPS holder using conductive carbon tape. The X-ray gun power was set to 72 W (6 mA and 12 kV). Survey scans were acquired using 200 eV pass energy, 0.5 eV step size, and 100 ms (50 ms × 2 scans) dwell times. Data analysis was performed using the Avantage and CASA XPS software packages. All samples were referenced against the C–C peak of adventitious carbon in the C 1s spectrum at a binding energy of 284.8 eV. For CNNS-1 and CNNS-2 deconvolution, a melon model was used.^32^

#### Textural, Structural, and Morphological Properties

The porosity of the samples was evaluated using N_2_ sorption at −196 °C following the same procedure as reported before.^26,33^ Specifically, the measurements were performed using a Micrometrics 3Flex sorption analyzer. A two-stage degas process was employed to evacuate the samples from any adsorbate traces. During the first stage, a Micromeritics VacPrep degasser was used to degas the samples overnight at 120° C and 0.2 mbar. The second stage consisted of an in situ degas at 110° C down to around 0.003 mbar for 4 h. The surface areas were calculated using the Brunauer–Emmett–Teller (BET) method (Table S3).^34^ The total volume of pores (*V*_TOT_) was calculated from the volume of N_2_ adsorbed at *P*/*P*_0_ = 0.97. The micropore volume (*V*_MICRO_) was determined using the Dubinin–Radushkevich method.^35^ The analyses were conducted on the powder samples as synthesized. Transmission electron imaging (TEM) images were taken by a JEOL 2100Plus instrument at an acceleration voltage of 200 kV, following the same procedure as reported before.^21^ Before imaging, the samples were sonicated in ethanol and then drop-casted on a carbon-coated copper grid.

#### Optoelectronic Properties

Valence band XPS and work function measurements were carried out on a Thermo Scientific K-Alpha^+^ X-ray photoelectron spectrometer equipped with a MXR3 Al Kα monochromated X-ray source (hν = 1486.6 eV) following the same procedure as reported before.^29,31^ Specifically, the samples were mounted on the XPS holder using conductive carbon tape. The X-ray gun power was set to 72 W (6 mA and 12 kV). Valence band spectra were obtained using 15 eV pass energy and 0.05 eV step size using the “area” mode (an average of 4 distinct points). Data analysis was performed using the software Thermo Avantage. The work functions of materials were determined by measuring the secondary electron cutoff in the low kinetic energy region. The sample holder contained a clean gold standard sample, which was used as a reference material to ensure correct calibration. A sample bias of −29.47 V was applied to the samples using an ion gun, and the cutoff spectra were obtained using a pass energy of 10 eV. To account for potential variations across the surface of the material, the work function was measured at three different locations and the average was taken. A total standard deviation of ±0.03 eV is associated with the band edge positions. To convert the valence band position and the work function to the absolute energy scale vs vacuum with the redox potential scale vs SHE, a shifting factor of +4.44 eV was required, as 4.44 eV on the former corresponds to 0.00 V on the latter, at 298 K. Diffuse reflectance ultraviolet–visible (DR-UV/Vis) spectra were obtained on the as-synthesized samples using a PerkinElmer Spectrum 100 spectrometer equipped with an integrating sphere following the same procedure as reported before.^26,29^ Specifically, spectral bandwidth was set to 2 nm, with Spectralon as a standard. Spectra were treated using the Kubelka–Munk function to eliminate any tailing contribution from the DR-UV–vis spectra. The following equation was applied: *F*(*R*) = (1 – *R*)^2^/2*R*, where *R* is the reflectance.^36^ The band gap (*E*_g_) values were estimated from the plot of *F*(*R*)hv^1/*n*^ vs energy by extrapolating the linear section. Such a plot is referred to as a Tauc plot. As Co-ZIF-L and Ti-MIL-125-NH_2_ are direct semiconductors, a value of *n* = 0.5 was used, while for CNNS (indirect semiconductor), a value of *n* = 2 was used.^37−39^ In the case of Co-ZIF-L, the determination of the band gap, which in turn influences the CB position, is not trivial. As shown in (Figure S3), Co-ZIF-L exhibits three features: ligand-to-metal charge transfer (LMCT), and two Co d–d transitions. Working on ZIF-67, the 3D counterpart of Co-ZIF-L, Pattengale et al. showed that upon photoexcitation, the electrons transition from the ground state to a Co d–d transition.^40^ In our case, to further confirm the participation of the d–d transitions, we measured the CO_2_ photoreduction activity of Co-ZIF-L using a >495 nm cutoff filter. The latter removes the photocatalytic contribution of the LCMT process occurring below 400 nm and allows us to investigate the photocatalytic contribution of the Co d–d transitions. As shown in Table S4, when using the cutoff filter, 40% of the “total” activity is retained, suggesting that the Co d–d transitions participate in CO_2_ conversion. Based on the obtained Co-ZIF-L Tauc plot, we use the d–d transition located ∼600 nm to determine the CB (Figure S4).

#### Gas Sorption Properties

CO_2_ sorption tests at low pressure (up to 1 bar) were performed on a Micromeritics 3Flex sorption analyzer at 25° C using a water bath to control the temperature following the same procedure as reported before.^29^ Prior to the analysis, the samples (∼80 mg) were degassed overnight at 120° C at roughly 0.2 mbar pressure and further degassed in situ for 4 h at 110° C down to around 0.0030 mbar, before the start of the analysis.

#### CO_2_ Photoreduction Testing

A gas/solid photoreactor was used to conduct CO_2_ photoreduction measurements at ambient temperature (Figure S5). Details of the photoreactor setup as well as the running of the experiments have been reported in our previous studies.^21,26,29,31,33,41^ For ease, we provide again these details here. The photocatalysts were deposited on a stainless steel plate with a fixed area of 9.6 cm^2^. To do so, 25 mg of the photocatalyst was dispersed in 1 mL of ethanol, sonicated for 30 s, and drop-casted onto the sample holder. Ethanol was evaporated, and the sample was activated overnight at 150° C in a vacuum oven. Research grade (99.999%) CO_2_ and H_2_ (99.9995%, Peak Scientific PH200 hydrogen generator) were flowed at controlled rates using mass flow controllers (Omega Engineering, 0–50 mL min^–1^). The photoreactor (35 cm^3^) was vacuumed and replenished with a gas mixture of CO_2_ and H_2_ (1.5 vol/vol ratio) five times. The same gas mixture of CO_2_ and H_2_ was subsequently passed over the catalyst bed in the photoreactor for 15 residence times to remove any impurities. The photoreactor was then sealed at 1.28 bar and irradiated for 5 h. A xenon arc lamp (300 W, λ > 325 nm, LOT Quantum Design) equipped with a water filter was used as the irradiation source. The distance from the lamp to the sample was 9.5 cm, with a broadband irradiance at the sample surface of 1830 W·m^–2^. Evolved gases were analyzed by a gas chromatograph (GC) with gas sampling valves connected directly to the photoreactor. The GC (Agilent Technologies, 7890B) was equipped with HayeSep (Agilent J&W 6 foot, 1/8 in., 2 mm, HayeSep Q Column 80/100 SST) and molecular sieve (Agilent J&W 6 foot, 1/8 in., 2 mm, MolSieve 5A, 60/80, preconditioned) packed columns in series, a thermal conductivity detector (TCD), and a flame ionization detector (FID). The photocatalytic CO_2_ reduction tests were repeated three times for each material under the same reaction conditions (except indicated otherwise). For recyclability tests, the aforementioned process was repeated after each 5 h irradiation cycle without opening the photoreactor. In addition, isotopic tracing experiments were performed with ^13^CO_2_ (BOC, >98%, ^13^CO_2_). The sample was placed in the photoreactor, vacuumed for 15 min, and subsequently flushed with He for 15 min. Afterward, a gas mixture of ^13^CO_2_ and H_2_ (1.5 vol/vol ratio) was passed over the catalyst bed for 3 min and pressurized at 1.28 bar. After irradiation, the evolved gases were analyzed by a mass spectrometer (Shimadzu MS) equipped with a Q-bond and a MolSieve 5A column with gas sampling valves connected directly to the photoreactor.

## Results and Discussion

### Characterization of CNNS/Co-ZIF-L Heterojunctions

**Figure 1:**
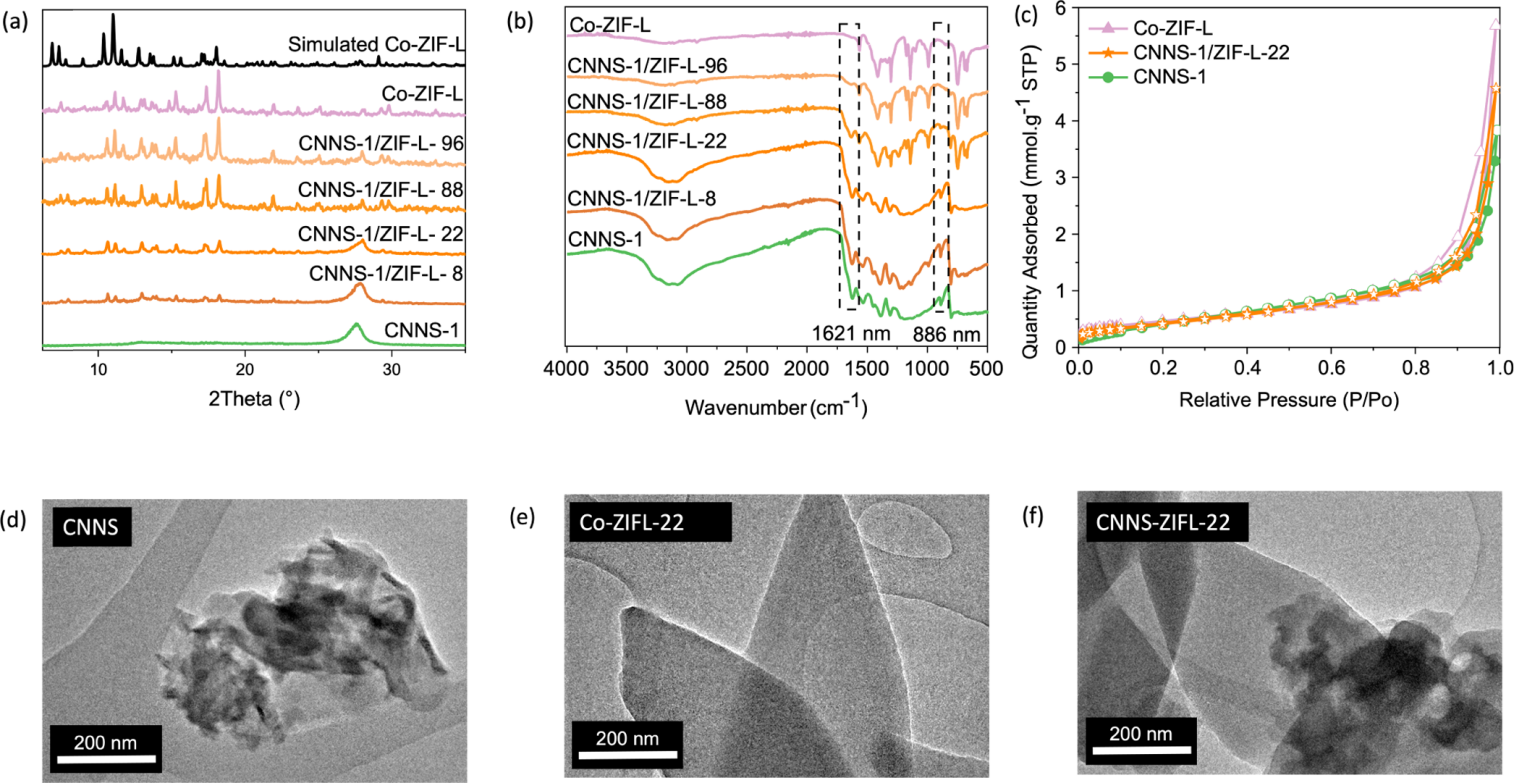
Chemical and structural characterization of the parent materials and CNNS-1/ZIF-L-*X* heterojunctions (where *X* refers to the weight percent of MOF used): (a) PXRD patterns; (b) ATR-FTIR spectra; (c) N_2_ sorption isotherms at 77 K; and (d–f) TEM images.

Using a one-pot solvothermal approach, we synthesized four different CNNS/Co-ZIF-L heterojunctions named CNNS-1/ZIF-L-*X*, where *X* represents the weight percent of MOF in the sample (*X* = 8, 22, 88, and 96 wt %, as determined by thermogravimetric analysis, Figure S1 and Table S1). A variety of characterization techniques confirmed the successful incorporation of both CNNS-1 and Co-ZIF-L in the heterojunctions. Figure 1a shows the XRD patterns of CNNS-1/ZIF-L-*X* exhibiting a combination of CNNS-1 and Co-ZIF-L patterns. Samples with a high MOF content show a high level of crystallinity with similar patterns to that of the pure MOF.^27^ Heterojunctions with a high CNNS-1 content exhibit the broad (002) peak of carbon nitride, corresponding to the spacing between stacked sheets.^13^ Similarly, ATR-FTIR spectra display bands from both CNNS-1 and Co-ZIF-L (Figure 1b). We attribute the broad vibrational band at 2800–3500 cm^–1^ to the stretching vibration of the N–H bond from the residual −NH_*x*_ group of CNNS-1.^42^ The 1627 and 1312 cm^–1^ region corresponds to C=N and C–N stretching modes, respectively, while the band at 804 cm^–1^ corresponds to the out-of-plane C–N bending of CNNS-1.^42,43^ The vibrational band at 1139 cm^–1^ is characteristic of the in-plane bending modes from the imidazole group present in Co-ZIF-L.^44^ Transmission electron microscopy (TEM) of CNNS-1 reveals an agglomerate of CNNS nanosheets forming clusters of ∼0.5 μm, while Co-ZIF-L displays a leaf-like morphology and a particle size of ∼2 μm. Finally, TEM images of CNNS-1/ZIF-L-22 display both Co-ZIF-L and CNNS-1 particles and show direct growth and contact between CNNS-1 and Co-ZIF-L, bringing further evidence of heterojunction formation (Figure 1d–f).

**Figure 2:**
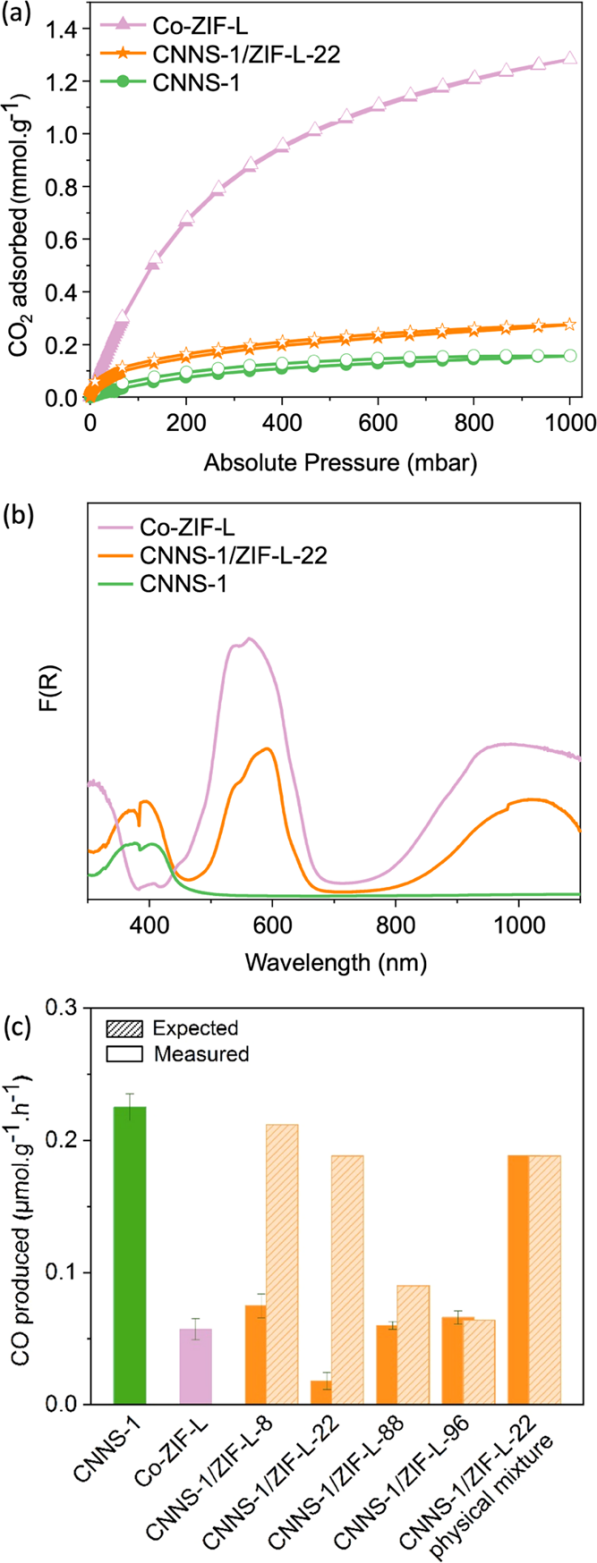
(a) CO_2_ sorption isotherms at 298 K, (b) UV–vis absorption spectra of CNNS-1, Co-ZIF-L, and CNNS-1/ZIF-L-22; (c) CO photocatalytic production rates of CNNS-1, Co-ZIF-L, and CNNS-1/ZIF-L-*X* heterojunctions (note: *X* corresponds to the weight percent of MOF used; error bars for CNNS-1/ZIF-L-88 and CNNS-1/ZIF-L-96 are based on two repeats and not three, as for the other samples).

Having confirmed the formation and composition of the heterojunctions, we next evaluated features that influenced their CO_2_ photoreduction properties, namely porosity, CO_2_ sorption, and light absorption. CNNS-1 displays a surface area and CO_2_ uptake at 298 K and 1 bar of 33 m^2^·g^–1^ and 0.15 mmol·g^–1^, respectively (Figures 1c and 2a and Table S3). The values for Co-ZIF-L are 41 m^2^·g^–1^ and 1.28 mmol·g^–1^ (Figures 1c and 2a and Table S3). As expected, considering the lower MOF content of CNNS-1/ZIF-L-22, the heterojunction displays a surface area of 37 m^2^·g^–1^ with a CO_2_ uptake of 0.27 mmol·g^–1^. Overall, compared to pure CNNS-1, the heterojunction formation favors higher CO_2_ uptakes due to the presence of Co-ZIF-L. The Tauc plots show that both CNNS-1 and Co-ZIF-L absorb UV and visible light with respective band gaps of 2.73 and 1.95 eV (Figure S5). As shown in Figure 2b, CNNS-1/ZIF-L-22 exhibits the absorption features of both Co-ZIF-L and CNNS-1.

### CO_2_ Photoreduction Activity and Band Bending Diagram of CNNS/Co-ZIF-L Heterojunctions

CO_2_ photoreduction tests were carried out in a heterogeneous gas/solid photoreactor at ambient temperature under UV–Vis irradiation (Xe arc lamp, 300 W, Figure S6) without the addition of a cocatalyst or photosensitizer. H_2_ was used as the sacrificial proton and electron source. All samples photoreduced CO_2_ with a 100% gaseous selectivity toward CO. To determine the effect of heterojunction formation on the CO_2_ photoreduction activity, we calculated an expected photoactivity based on the composition of the heterojunction components. In the absence of interaction, it would be reasonable to expect the activity of the composite to be proportional to the activity of the base materials. For example, for CNNS-1/ZIF-L-22, composed of 22 wt % MOF and 78 wt % CNNS-1 (see Table S1), the expected activity is the sum of Co-ZIF-L photoactivity multiplied by 0.22 and CNNS-1 photoactivity multiplied by 0.78. We then compared these activity values to the activity actually measured for the composites (Figure 2c and Table S4). Surprisingly, the formation of a CNNS-1/ZIF-L heterojunction negatively impacts the photoreduction activity in most cases. As shown in Figure 2c, for CNNS-1/ZIF-L-96, the activity remains unimproved, while for CNNS-1/ZIF-L-8, -22, and -88, the amount of CO produced decreases upon heterojunction formation. For heterojunctions with a high MOF content, the activity resembles one of the physical mixtures, whereas, for the others, the heterojunction formation negatively impacts the activity. Such observation corroborates that the intimate contact between CNNS-1 and Co-ZIF-L negatively impacts the photoreduction activity. Such behavior is most noticeable for CNNS-1/ZIF-L-22, where CO production is 0.018 μmol·g^–1^·h^–1^ compared to 0.188 μmol·g^–1^·h^–1^ for the expected value.

To further establish the effects of CNNS-1/ZIF-L-22 heterojunction formation on photoactivity, we prepared and tested a physical mixture containing the same composition as the latter (Figure 2c and Table S4). The activity of the physically mixed sample is close to the expected value (0.189 μmol·g^–1^·h^–1^ vs 0.188 μmol·g^–1^·h^–1^, respectively), whereas we observed a 10-fold decrease in the activity between the physical mixture and the in situ synthesized heterojunction. Given that both components have some degree of inherent activity for CO_2_ reduction, these results show that the intimate contact between CNNS-1 and Co-ZIF-L upon heterojunction formation negatively impacts the CO_2_ photoreduction activity. In the literature, numerous reports show an enhanced CO_2_ photoreduction activity in MOF-based heterojunctions.^18,21,41^ Yet, studies do not commonly focus on investigating and understanding the origin of enhancements (or losses) in MOF-based heterojunctions.

**Figure 3:**
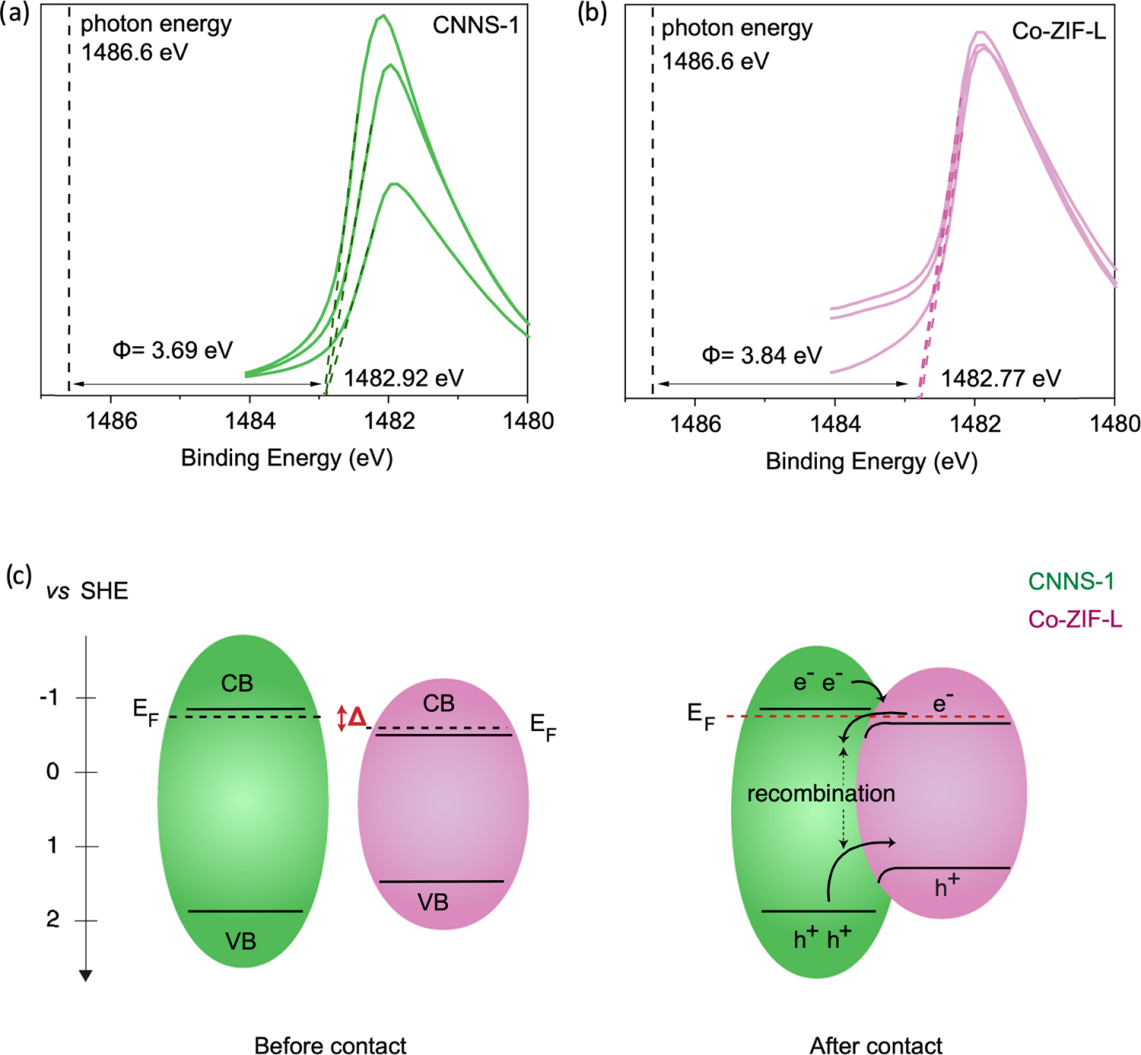
Work function measurements of: (a) CNNS-1 and (b) Co-ZIF-L; (c) band bending diagram of CNNS-1/Co-ZIF-L heterojunction before and after contact.

To understand the photochemical behavior of our system—i.e., how electrons flow across the heterojunction—we first resolved the electronic structure of CNNS-1 and Co-ZIF-L (Figures 3c and S7–S9). While doing so is common in the literature for MOF-based heterojunctions, the obtained electronic structures do not usually consider the effects of band bending and band alignment.^45^ Heterojunction systems are better described by band bending diagrams.^46^ This concept is widespread in the photoelectrochemisty literature but much less so in MOF photocatalysis studies.^45−47^ Band bending occurs when two semiconductors with distinct work functions—i.e., different Fermi levels—are put into contact. Upon contact, electrons transfer from one semiconductor to the other until equilibrium is reached, i.e., both Fermi levels are aligned. This spontaneous electron diffusion favors the formation of negative and positive charges at the interface, generating an internal electric field. This electric field results in upward or downward band bending. Band bending upon heterojunction formation can thus affect how charge carriers flow across the heterojunction, thereby influencing electron–hole recombination/separation. While electronic structures for MOF-based heterojunctions tend to neglect band bending and band alignment, investigating these effects enables one to better describe the photocatalytic process occurring at a heterojunction interface. This concept appears in the recently introduced step-scheme (S-scheme) MOF-based heterojunction; however, it is usually not considered in other (i.e., non S-scheme) MOF-based heterounctions.^23,24^ To understand the electron flow in our system, we determined the band alignment using XPS and used this information to produce a band bending diagram for the CNNS-1/Co-ZIL-L heterojunctions (Figures 3c and S10–S14). We discuss the implication of our findings below.

### Importance of Band Bending and Band Alignment to Understand Electron Flow in MOF-Based Heterojunctions

Using XPS, we investigated the surface chemical states of CNNS-1, Co-ZIF-L, and CNNS-1/ZIF-L-22 (Figures S10–S12). The main C 1s peak of CNNS-1 at 288 eV corresponds to sp^2^ bonded carbons (N=C–N), while the N 1s core-level spectrum displays four deconvoluted peaks at 398.6, 399.3, 400.2, and 401.2 eV, characteristic of the heptazine ring, heptazine core, −N–H_2,_ and N–H groups, respectively.^48,49^ Regarding Co-ZIF-L, the deconvoluted Co 2p spectrum shows eight different binding energies at 781.8, 784.1, 797.2, and 799.4 eV, attributed to Co^3+^ 2p_3/2_, Co^2+^ 2p_3/2_, Co^3+^ 2p_1/2_, Co^2+^ 2p_1/2_ respectively, along with the satellite peaks at 786.9, 789.7, 802.7, and 805.0 eV.^50^,^51^ The main C 1s peaks correspond to C–C (284.8 eV), C–CH_3_ (285.0 eV), N–C–C–N (285.6 eV), and C–N–C (286.6 eV) bonds, while the peaks at 398.9 and 400.6 eV in the N 1s spectrum relate to the sp^2^ and sp^3^ nitrogen of the imidazole linker.^50^

Interestingly, compared to Co-ZIF-L, in CNNS-1/ZIF-L-22, a 0.8 eV shift to higher binding energy is observed in the Co 2p and O 1s core levels associated with Co-ZIF-L (Figures S13 and S14). This shift is unlikely the result of identical chemical shifts for both Co 2p and O 1s core levels. Instead, we attribute these changes to a Fermi level shift.^47,52,53^ Since the Fermi level of a material is defined at 0 eV in XPS, a constant shift of all Co-ZIF-L core line peaks toward higher binding energy suggests an upward shift in the Fermi level position upon heterojunction formation.^21,52,53^

As defined by eqs S1 and S2 in Figure S14, the difference in binding energy between two peaks is independent of the position of the Fermi level. Figure S14 shows that, indeed, the separation of the Co 2p and O 1s peaks remains constant in Co-ZIF-L and CNNS-1/ZIF-L-22, further suggesting it corresponds to a Fermi level shift. We did not observe any significant shift in the core level associated with CNNS-1, indicating the absence of a Fermi level shift and, therefore, the absence of band bending on CNNS-1. We foresee that the potential drop generated by interfacial charge transfer may mainly distribute across the MOF. Hence, upon heterojunction formation, electrons transfer from CNNS-1 to Co-ZIF-L until the Fermi levels align. This upward shift is consistent with the sign of the work function difference between CNNS-1 and Co-ZIF-L (i.e., 3.69 eV vs 3.84 eV, respectively, Figure 3a,b). Overall, upon heterojunction formation, we interpret the change in the position of the Fermi level to be equal to the potential drop in the heterojunction upon equilibration of the two materials forming the junction. In semiconductor-based heterojunctions, the change in chemical potential upon heterojunction formation can be used to probe band bending. An upward shift of the Fermi level upon interface contact suggests downward band bending and vice versa. Based on these results and using band gap, valence band, and work function measurements, we built the band bending diagram, as shown in Figure 3c.

According to Figure 3c, after heterojunction formation, a Type I straddling gap is formed. Under illumination, the photogenerated electrons of CNNS-1 are predicted to migrate to the conduction band (CB) of Co-ZIF-L. The straddling junction combined with downward band bending will likely cause electrons to accumulate in Co-ZIF-L at the heterojunction interface with CNNS-1. This could hinder electrons from flowing to the surface of Co-ZIF-L, where they can reduce CO_2_. In addition, such accumulation of electrons at the heterojunction interface may reduce their mobility and increase the likelihood of recombination with the photogenerated holes that also accumulate in Co-ZIF-L. We note that this band bending diagram is in agreement with the observed photocatalytic results, suggesting a decrease of available photogenerated charges upon heterojunction formation.

### Characterization of CNNS/Ti-MIL-125-NH_2_ Heterojunction

**Figure 4:**
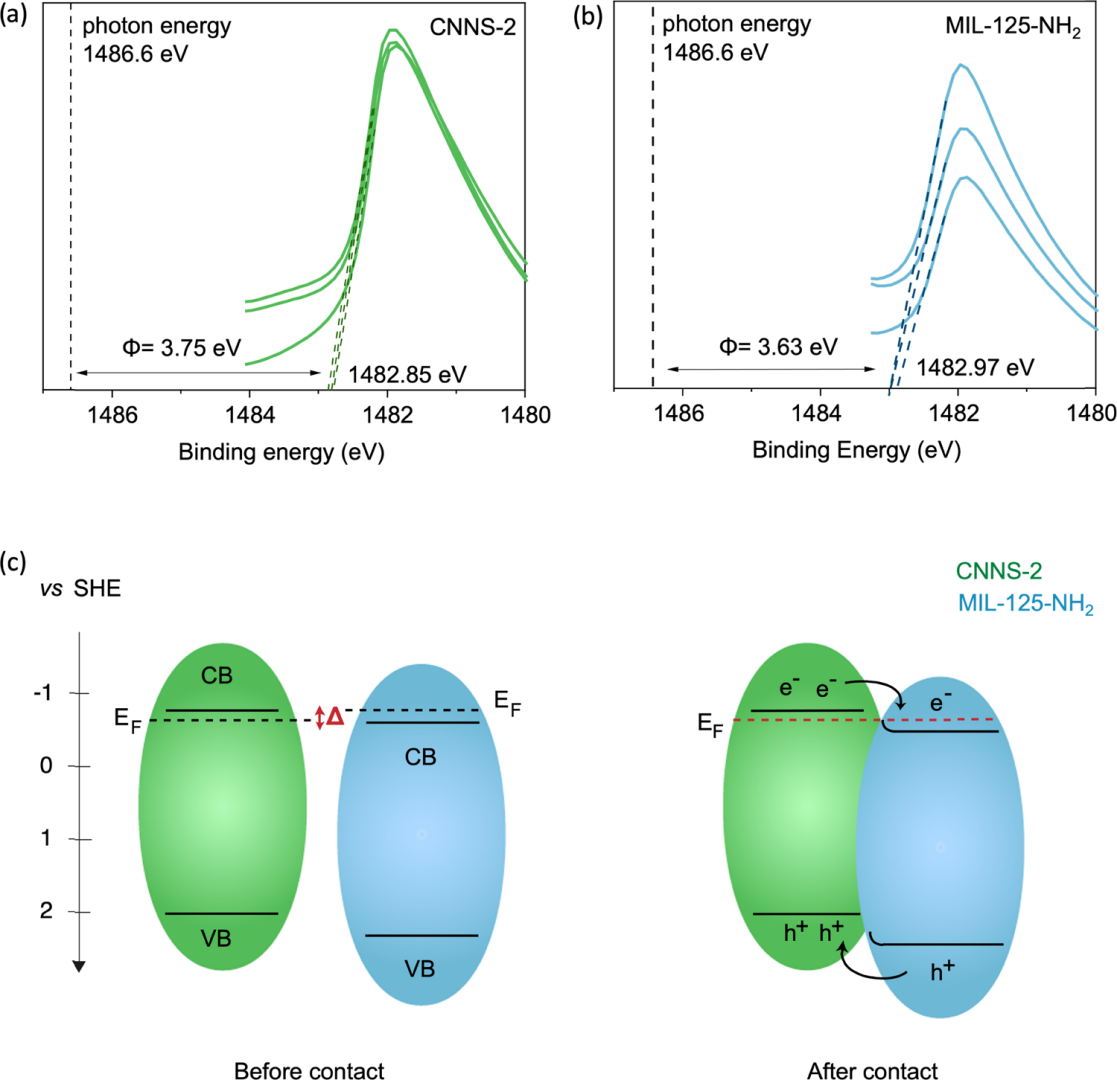
Work function measurements of: (a) CNNS-2 and (b) MIL-125-NH_2_; (c) band bending diagram of CNNS-2/MIL-125 heterojunction before and after contact.

**Figure 5:**
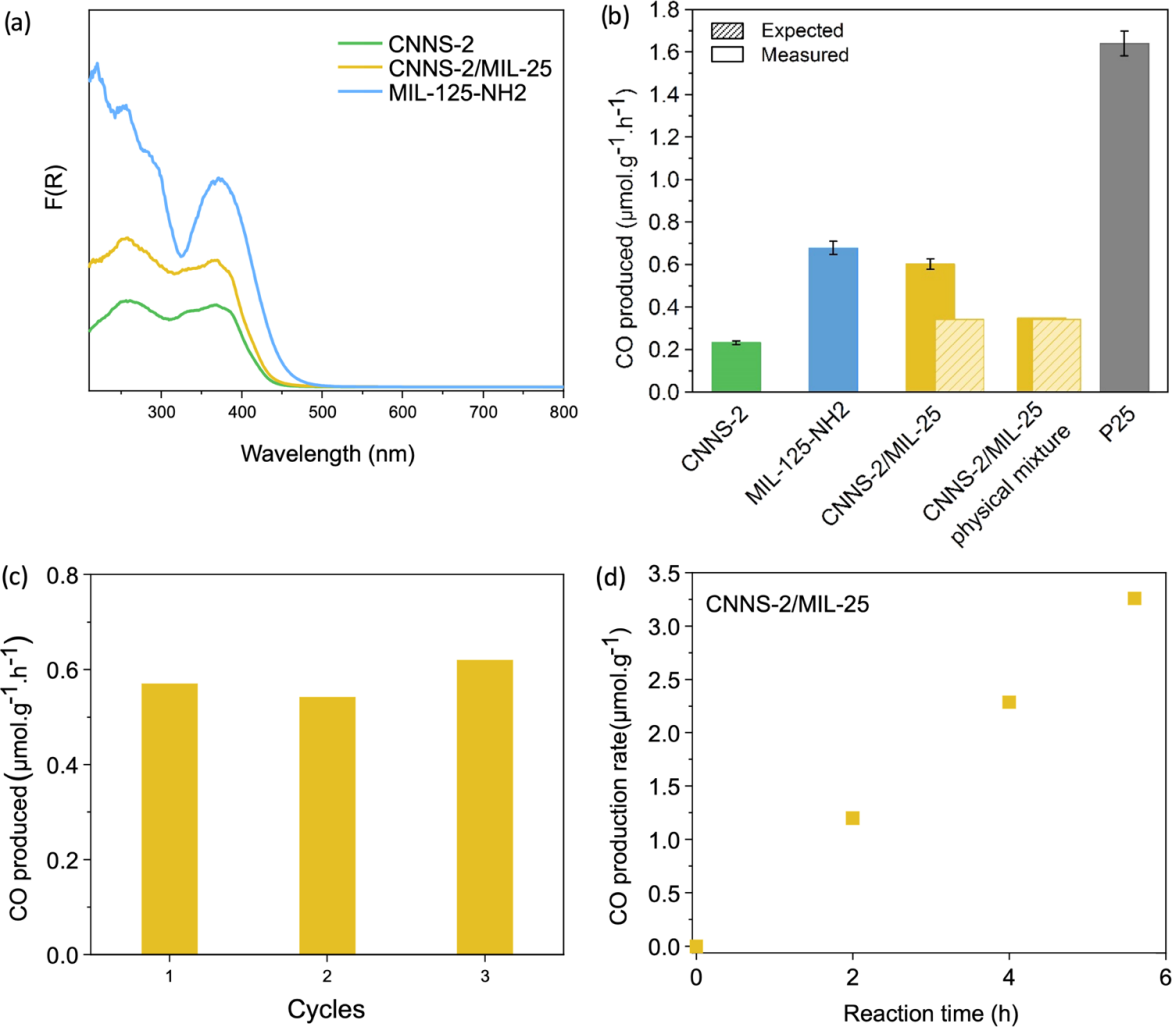
(a) UV–vis absorption spectra of CNNS-2, MIL-125-NH_2_, and CNNS-2/MIL-25, (b) photocatalytic CO evolution rate of CNNS-2, MIL-125-NH_2_, and CNNS-2/MIL-25 heterojunction under UV–vis irradiation; (c) CO evolution rates after repeated photocatalytic measurements, and (d) kinetic study of CO_2_ photoreduction using CNNS-2/MIL-25.

Based on the above findings, we developed an alternative CNNS/Ti-MIL-125-NH_2_ heterojunction for CO_2_ photoreduction with a more favorable band bending diagram. The synthesized CNNS/Ti-MIL-125-NH_2_ heterojunction is named CNNS-2/MIL-25, where the number 25 represents the weight percent of MOF in the sample, as determined by thermogravimetric analysis (Figure S2 and Table S2). For the composites based on Ti-MIL-125-NH_2_ synthesis, we used another batch of CNNS, namely CNNS-2. Both CNNS-1 and CNNS-2 exhibit comparable physicochemical properties with similar band edge position and band gaps (i.e., 2.73 and 2.75 eV, respectively, Figures 3, 4, S5, and S15). In a similar manner to CNNS-1/Co-ZIF-L, PXRD, ATR-FTIR, TEM, UV–vis absorption, N_2_ adsorption isotherms at 77 K, and CO_2_ adsorption isotherms at 298 K measurements confirmed the successful formation of a CNNS-2/MIL-125-NH_2_ heterojunction (Figures S16–S19 and Table S5). We then conducted UV–Vis DRS analyses, core-level XPS analyses, and valence band and work function measurements to determine the band bending diagram of CNNS-2/MIL-25 heterojunction. Both CNNS-2 and MIL-125-NH_2_ absorb UV and visible light with respective band gaps of 2.75 and 2.93 eV (Figures 5a and S15). The conduction band and valence bands of CNNS-2 and MIL-125-NH_2_ were located at −0.79, 1.96 eV and −0.4, 2.53 eV, respectively, in agreement with values reported in the literature (Figures S15, S20, and S21 and Table S6).^37,54,55^ We determined the work functions of CNNS-2 and MIL-125-NH_2_ to be at 3.75 and 3.63 eV, respectively (Figure 4a,b).

High-resolution XPS shows C 1s, N 1s core levels of CNNS-2 and Ti 2p, C 1s, N 1s, and O 1s core levels of MIL-125-NH_2_ in line with reported literature (Figures S22–S24).^26,42,56^ Deconvolution of the C 1s spectra of MIL-125-NH_2_ shows four different binding energies at 298.9, 286.8, 285.2, and 284.8 eV attributed to C=O, C–N, C=C, and C–C, respectively. Ti 2p displays two binding energy peaks at 458.9 and 464.6 eV, attributed to Ti 2p_3/2_ and Ti 2p_1/2_ of Ti^IV^, respectively.^57^ For the oxygen environment, we observed two peaks at 532.7 and 531.2 eV, corresponding to C=O and titanium oxo clusters.^56^

Compared to MIL-125-NH_2_, In CNNS-2/MIL-25, a shift to lower binding energy is observed in the Ti 2p and O 1s core levels associated to MIL-125-NH_2_ (Figure S25). As for CNNS/ZIF-L, we observe a similar chemical shift for both Ti 2p and O 1s core levels in CNNS-2/MIL-25. Additionally, we see a constant separation of these two core levels in MIL-125-NH_2_ and CNNS-2/MIL-25, suggesting that the observed shift corresponds to a Fermi level shift (Figure S26). Similarly to CNNS/Co-ZIF-L, we did not observe any significant shift in the core level associated to CNNS-2, suggesting that no band bending is occurring on CNNS-2. We foresee that the potential drop generated by interfacial charge transfer may mainly distribute across the MOF. In addition, this downward shift is consistent with the sign of the work function difference between CNNS-2 and MIL-125-NH_2_ (i.e., 3.75 eV vs 3.63 eV, respectively, Figure 4a,b). Upon heterojunction formation, electrons will transfer from MIL-125-NH_2_ to CNNS-2 until MIL-125-NH_2_ Fermi level aligns with that of CNNS-2. Based on these results and using band gap, valence band, and work function measurements, we constructed the band bending diagram, as shown in Figure 4c. After contact and under illumination, photogenerated electrons migrate to MIL-125-NH_2_, while holes migrate to CNNS-2. In this Type II heterojunction, charges should be spatially separated and heterojunction formation should favor higher photoactivity.

To verify this hypothesis, CO_2_ photoreduction tests were performed on CNNS-2, MIL-125-NH_2_, and CNNS-2/MIL-25. The photocatalytic CO_2_ reduction tests were repeated three times for each material under the same reaction conditions. To determine the effect of heterojunction formation on the CO_2_ photoreduction activity, we calculated an expected photoactivity based on the contribution of the heterojunction components as defined for CNNS-1/ZIF-L-*X* (Figure 5b and Table S7). In addition, we prepared and tested a physical mixture containing the same composition of CNNS-2/MIL-25. All samples photoreduced CO_2_ with a 100% gaseous selectivity toward CO. As Figure 5b shows, CNNS-2 produces 0.23 ± 0.009 μmol·g^–1^·h^–1^ of CO, while MIL-125-NH_2_ produces 0.68 ± 0.031 μmol·g^–1^·h^–1^. After heterojunction formation, the CNNS-2/MIL-25 heterojunction shows a 1.8-fold photocatalytic improvement compared to the physical mixture and the expected photoactivity (i.e., 0.60 ± 0.025 μmol·g^–1^·h^–1^ vs 0.35 and 0.34 μmol·g^–1^·h^–1^, respectively). These results highlight the positive impact of an intimate contact between CNNS-2 and MIL-125-NH_2_ upon heterojunction formation that leads to enhanced photoactivity. Based on the band bending diagram, we attribute this increase of activity to an enhanced electron/hole separation made possible by the formation of a Type II staggered gap heterojunction. The band bending found in this heterojunction results in a thermodynamic driving force that drives charge carrier separation, with photogenerated electrons and holes flowing into MIL-125-NH_2_ and CNNS-2, respectively. The adequate band alignment and band bending led to an enhanced photoactivity, which could be the result of improved CO_2_ reduction and/or H_2_ oxidation. Finally, the performance of CNNS-2/MIL-25 was compared to the benchmark heterojunction P25 TiO_2_, composed of ∼80% anastase and ∼20% rutile TiO_2_, which showed a remarkably higher activity of ∼3-fold.

To verify the photocatalytic production of CO from CO_2_, we conducted a series of control experiments (Table S8). In the absence of CO_2_ (i.e., N_2_/H_2_ environment), the activity decreased by 73%. Additionally, we performed isotopic labeled ^13^CO_2_ measurement. As shown in Figure S27, after light irradiation, we observed a ^13^CO peak (*m*/*z* = 29), confirming the photocatalytic origin of the detected CO. Kinetic studies point to a linear production of CO during 5 h and 40 min of irradiation (Figure 5d). In addition, recyclability tests indicate no decline in the photocatalytic performance over three cycles (Figure 5c). CNNS-2/MIL-25 does not dramatically lose its activity after three cycles. Yet, to assess its photocatalytic stability, one needs to perform post-reaction characterization. To do so, we collected XRD spectra, high-resolution XPS, N_2,_ and CO_2_ adsorption isotherms before and after photocatalytic testing. As Figures S28–S30 show, adsorption isotherms and high-resolution XPS spectra of C 1s, N 1s, O 1*s*, and Ti 2p core levels did not change significantly, pointing to a relatively high photocatalytic stability. Having confirmed the photocatalytic nature of CNNS-2/MIL-25, we conclude that contrary to CNNS-1/Co-ZIF-L heterojunction, for which band bending and band alignment appear to inhibit the photoactivity compared to the physical mixture, band bending effects in CNNS-2/Ti-MIL-125-NH_2_ enhanced photoactivity by favoring a Type II heterojunction, which more efficiently separates photogenerated charge.

## Conclusions

In this study, we demonstrate how considering band bending and band alignment enables us to rationalize the photocatalytic enhancements or losses in MOF-based heterojunctions compared to their individual components. Using the examples of CNNS/Co-ZIF-L and CNNS/Ti-MIL-125-NH_2_ heterojunctions for CO_2_ photoreduction, we show how to construct a simple band model that can predict the positive or negative impact of band bending on the photoreduction activity. Band bending effects can inhibit or enhance CO_2_ photoreduction activity, depending on the heterojunction components. Therefore, when developing a heterojunction, the choice of the heterojunction components is important and one should establish a band bending diagram at an early stage of the research, an aspect that is not commonly investigated for MOF-based heterojunctions. Investigating the effects of band bending becomes particularly important when the heterojunction involves two semiconductors with distinct work functions. Here, based on favorable band alignment and band bending, we developed a Type II CNNS/Ti-MIL-125-NH_2_ heterojunction that promoted the spatial separation of electrons and holes. The adequate band alignment and band bending led to an enhanced apparent photoactivity, which could be the result of improved CO_2_ reduction and/or H_2_ oxidation. Specifically, this heterojunction showed selective CO_2_ photoreduction to CO with a 1.8-fold photocatalytic improvement compared to the physical mixture. Despite the improvements in CNNS-2/MIL-25 photocatalytic activity, the activity remains lower than the photocatalytic benchmark TiO_2_ P25 heterojunction. Future studies should focus on developing new MOF-based heterojunctions with even more favorable band alignment and band bending that would confer higher performance than this benchmark material.

## Supporting Information Available

Additional experimental details, TGA, Tauc plot, valence band, high-resolution XPS, TEM images, XRD, FTIR, UV–vis, and ^13^CO_2_ tests (PDF)

## Abbreviations

CNNS: graphitic carbon nitride nanosheets
2-Hmin: 2-methylimidazole
