# Supplementary material for: Effect of Band Bending in Photoactive MOF-Based Heterojunctions
Source: ACS Appl Mater Interfaces. 2022 Apr 20;14(17):19342–52. doi: 10.1021/acsami.2c00335 (PMC9073837; doi:10.1021/acsami.2c00335)
Supplement: Supplementary file 1 — am2c00335_si_001.pdf [file am2c00335_si_001.pdf]

Supporting Information

The effect of band bending in photoactive MOF-based heterojunctions

Giulia E. M. Schukraft,[a,b] Benjamin Moss,[c] Andreas G. Kafizas,[c,d] and Camille Petit[a*]

[a] *Barrer Centre, Department of Chemical Engineering, South Kensington Campus, Imperial College London, London, SW7 2AZ, UK*

[b] *Department of Materials, South Kensington Campus, Imperial College London, London, SW7 2AZ, UK*

[c] *Department of Chemistry, Molecular Science Research Hub, White City Campus, Imperial College London, London, W12 0BZ, UK*

[d] *The Grantham Institute, Imperial College London, London SW7 2AZ, United Kingdom*

*Corresponding author: camille.petit@imperial.ac.uk

Table of Contents

1. Supplementary Figures and Tables 3
2. References 41

1. Supplementary Figures and Tables

Table S1. Composition of the samples as derived by the thermogravimetric analysis of CNNS-1, Co-ZIF-L and CNNS-1/ZIF-L-X heterojunctions. The numbers in parenthesis correspond to approximations to the nearest unit.

**Samples**	**Final mass (% mass)**	**Mass loss**[a] **(% mass)**	$\mathbf{X_{CNNS\text{-}1}}^{b}$ **(% mass)**	$\mathbf{X_{Co\text{-}ZIF\text{-}L}}^{c}$ **(% mass)**
Co-ZIF-L	48.57		0	100
CNNS-1/ZIF-L-96	46.78	1.79	3.68 (4)	96
CNNS-1/ZIF-L-88	42.76	5.81	11.96 (12)	88
CNNS-1/ZIF-L-22	10.79	37.78	77.78 (78)	22
CNNS-1/ZIF-L-8	3.68	44.71	92.42 (92)	8
CNNS-1	0.08		100	0

[a]. $\text{Mass loss} = \text{Co - ZIF - L}_{\text{Final mass}} - \text{CNNS - 1/ZIF - L - X}_{\text{Final mass}}$

[b]. Calculated: $X_{\text{CNNS}-1} = \frac{\Delta\text{mass loss}}{\text{Co}-\text{ZIF}-\text{L}_{\text{Final mass}}}$

[c]. $X_{\text{Co}-\text{ZIF}-\text{L}} = 1 - X_{\text{CNNS}-1}$

Table S2. Composition analysis of CNNS-2, MIL-125-NH_2 and CNNS-2/MIL-25 derived from thermogravimetric analyses. The number in parenthesis correspond to approximations to the nearest unit.

Samples	Final mass (% mass)	Mass loss[a] (% mass)	x_{CNNS-2}[b] (% mass)	$x_{MIL-125-NH2}$[c] (% mass)
MIL-125-NH_2	53.69		0	100
CNNS-2/MIL-25	13.71	39.98	74.45 (75)	25
CNNS-2	0.2		100	0

[a]. Calculated: $\text{Mass loss} = \text{MIL - 125 - NH2}_{\text{Final mass}} - \text{CNNS - 2/MIL - 25}_{\text{Final mass}}$

[b]. Calculated: $X_{\text{CNNS}-2} = \frac{\Delta\text{mass loss}}{\text{MIL} - 125 - NH_{2\,\text{Final mass}}}$

[c]. $X_{\text{MIL}-125-\text{N}H_2} = 1 - X_{\text{CNNS}-2}$

Table S3. Summary of textural properties derived from N_2 sorption isotherms at 77 K and CO_2 adsorption capacity at 298 K and 1 bar of CNNS-1, Co-ZIF-L and CNNS-1/ZIF-L-22.

**Sample**	**V_{MICRO} (cm³/g)**	**V_{TOT} (cm³/g)**	**S_{BET} (m²/g)**	**CO_2 ads. (mmol/g)**
CNNS-1	nil	0.049	33	0.15
Co-ZIF-L	nil	0.053	41	1.28
CNNS-1/ZIF-L-22	nil	0.051	37 (35)[a]	0.28 (0.4)[b]

[a] Expected value $= (S_{BET\ CNNS\text{-}1} \times 0.78) + (S_{BET\ Co\text{-}ZIF\text{-}L} \times 0.22)$

[b] Expected value $= (CO_2\ ads._{CNNS\text{-}1} \times 0.78) + CO_2\ ads._{Co\text{-}ZIF\text{-}L} \times 0.22)$

Table S4. CO evolution rates for CNNS-1, Co-ZIF-L and CNNS-1/ZIF-L-X photocatalysts under UV-vis irradiation using H_2 as reducing agent.

**Samples**	**CO produced (μmol.g^{-1}.h^{-1})**	**Expected photoactivity[a] (μmol.g^{-1}.h^{-1})**
Co-ZIF-L	0.057 ± 0.008<br>0.023 (λ > **495nm)**	
CNNS-1	0.22 ± 0.01	
CNNS-1/ZIF-L-96	0.066 ± 0.005	0.064
CNNS-1/ZIF-L-88	0.060 ± 0.003	0.09
CNNS-1/ZIF-L-22	0.018 ± 0.01	0.188
CNNS-1/ZIF-L-22 physical mixture	0.189	0.188
CNNS-1/ZIF-L-8	0.075 ± 0.009	0.21

[a] Expected value $= (\text{activity}_{\text{CNNS}} \times \text{X}_{\text{CNNS}}) + (\text{activity}_{\text{MOF}} \times \text{X}_{\text{MOF}})$, where X represents the weight percent of CNNS or MOF in the sample.

Table S5. Summary of textural properties derived from the N_2 sorption isotherms at 77 K and CO_2 adsorption capacity at 298 K and 1 bar of CNNS-2, MIL-125-NH_2 and CNNS-2/MIL-25.

Sample	V_{MICRO} (cm^3/g)	V_{TOT} (cm^3/g)	S_{BET} (m^2/g)	CO_2 ads. (mmol/g)
CNNS-2	nil	0.16	32	0.13
MIL-125-NH_2	0.55	0.67	1635	3.64
CNNS-2/MIL-25	0.13	0.37	462 (433 expected)[a]	1.07 (1.00 expected)[b]
CNNS-2/MIL-25 after irradiation	0.11	0.34	403	0.98

[a] Expected value $= (S_{BET\ CNNS-2} \times 0.25) + (S_{BET\ MIL-125-NH2} \times 0.75)$

[b] Expected value $= (CO_2\ ads._{CNNS\text{-}2} \times 0.25) + CO_2\ ads._{MIL-125-NH2} \times 0.75)$

Table S6. Comparison of the measured band edge position of CNNS, MIL-125-NH_2 and ZIF-67 with those reported in literature.

Photocatalyst			Method used	Ref
CNNS	-0.78 eV	2.07 eV	Mott–Schottky	[1]
CNNNS	-0.67 eV	2.0 eV	Mott–Schottky	[2]
CNNS	-0.75 eV	2.05 eV	XPS	[3]
CNNS-1	-0.85 eV	2.0 eV	XPS	This work
CNNS-2	-0.79 eV	1.96 eV	XPS	This work
MIL-125-NH_2	-0.24	2.45	Mott–Schottky	[4]
MIL-125-NH_2	-0.43	2.34	Mott–Schottky	[5]
MIL-125-NH_2	-0.40	2.53	XPS	This work
ZIF-67	-0.45 eV	1.39	Mott–Schottky	[6]
ZIF-67	-0.74 eV	1.18	Mott–Schottky	[7]
Co-ZIF-L (2D ZIF-67)	-0.72	1.23	XPS	This work

Table S7. CO evolution rates for CNNS-2, MIL-125-NH_2, CNNS-2/MIL and P25 TiO_2 photocatalysts under UV-vis irradiation using H_2 as reducing agent.

**Samples**	**CO produced ($\mu mol.g^{-1}.h^{-1}$)**	**Expected photoactivity[a] ($\mu mol.g^{-1}.h^{-1}$)**
Ti-MIL-125-NH_2	0.68 ± 0.03	
CNNS-2	0.23± 0.009	
CNNS-2/MIL-25	0.60 ± 0.025	0.34
CNNS-2/MIL-25 physical mixture	0.35	0.34
P25 TiO_2	1.64 ± 0.058	

[a] Expected value=(activity $_{CNNS}$ × $X_{\_CNNS}$) + (activity$_{_MOF}$ × $X_{\_MOF}$), where X represents the weight percent of CNNS or MOF in the sample.

Table S8. CO_2 photocatalytic control experiments. The former two tests were performed on CNNS-2/MIL-25, the leading material of this study using H_2 as reducing agent. UV-vis irradiation was used when needed.

**Samples**	**CO produced (μmol.g^{-1}.h^{-1})**
N_2/H_2 (catalyst + irradiation)	0.17 ± 0.013
N_2/H_2 (catalyst + no irradiation)	nil
N_2/H_2 (no catalyst + irradiation)	nil

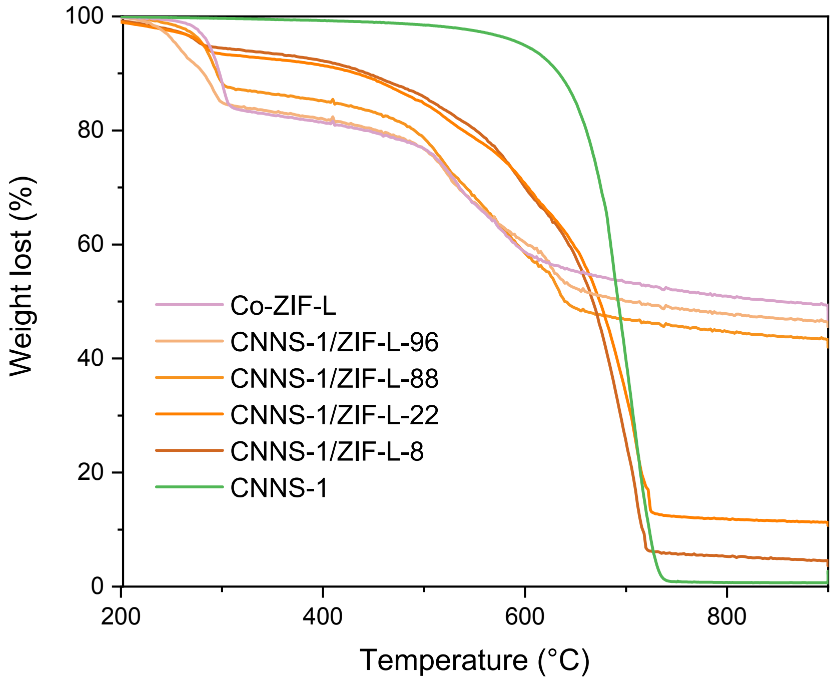

Figure S1. Thermogravimetric analyses of CNNS-1, Co-ZIF-L and CNNS-1/ZIF-L-X heterojunctions under N_2 atmosphere.

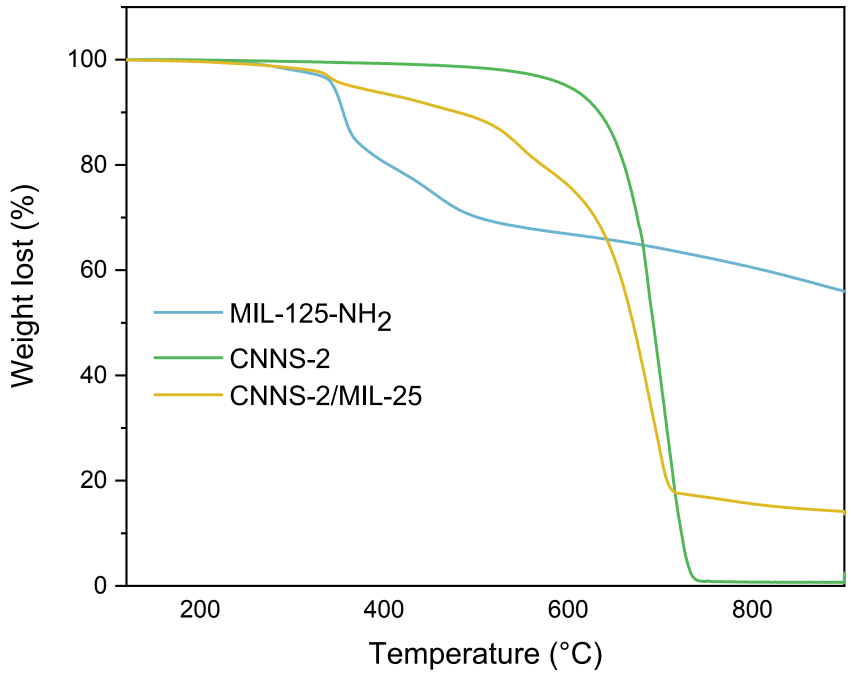

Figure S2. Thermogravimetric analysis of CNNS-2, MIL-125-NH_2 and CNNS-2/MIL-25 under N_2 atmosphere.

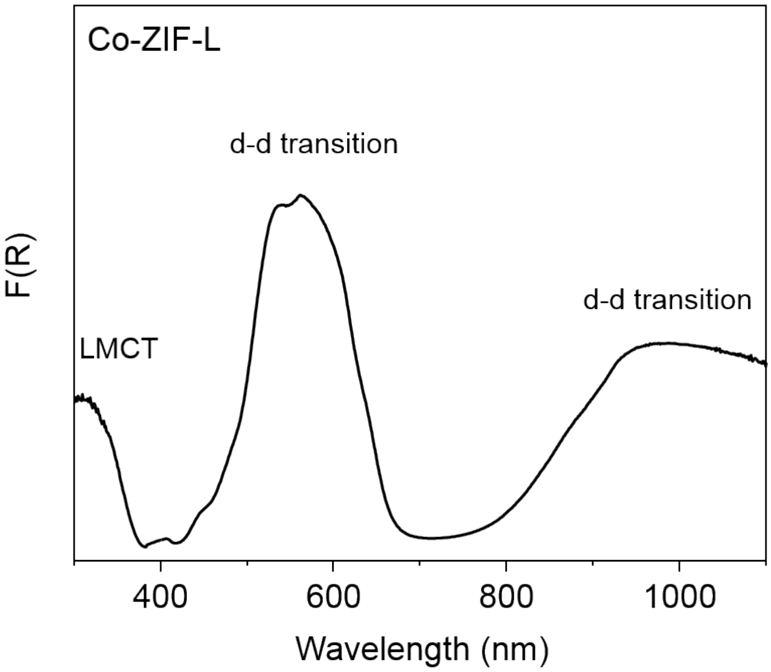

Figure S3. UV-vis absorption spectra of Co-ZIF-L showing the absorption bands of the ligand-to-metal charge-transfer (LMCT) and two Co d-d absorptions bands.

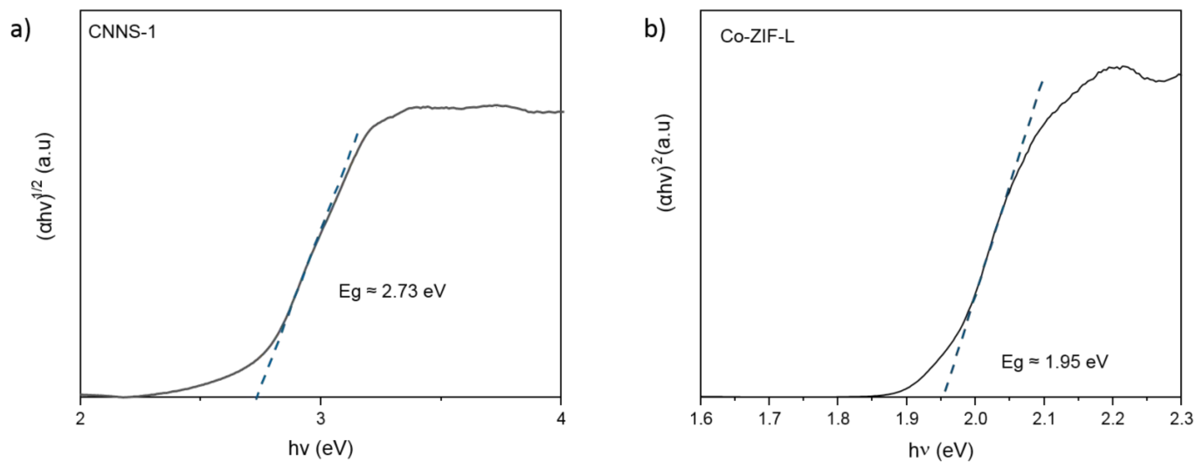

Figure S4. Tauc plots of a) CNNS-1 and b) Co-ZIF-L. The UV-vis reflectance spectra were converted to Tauc plots using the transformed Kubelka-Munk function.[1,2]

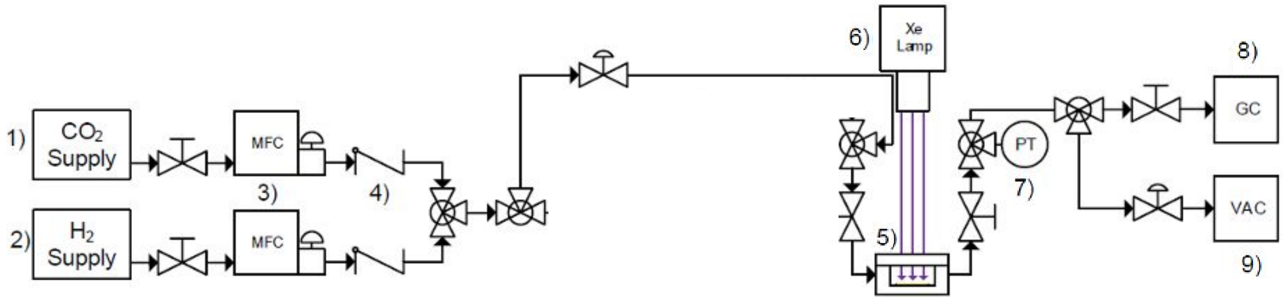

Figure S5. Photocatalytic gas-solid reactor setup used to evaluate photocatalytic CO_2 reduction: 1) CO_2 cylinder, 2) H_2 generator, 3) mass flow controllers, 4) non-return valves, 5) photoreactor, 6) Xe arc lamp, 7) pressure transducer, 8) gas chromatograph, 9) vacuum pump. Adapted from ref [3].

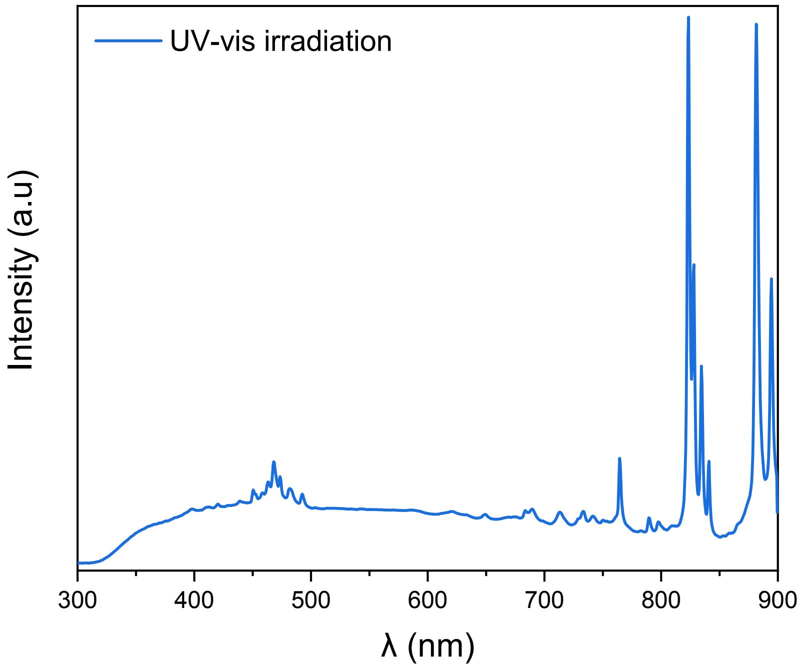

Figure S6. Xenon arc lamp emission spectra, (300W, LOT Quantum Design), equipped with a water filter.

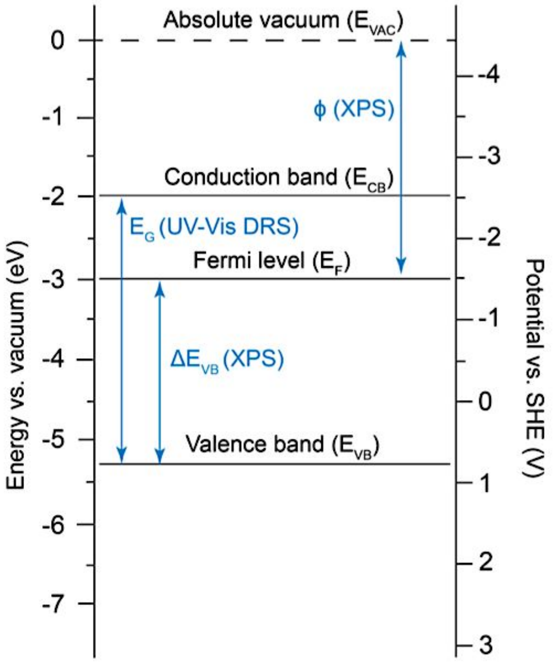

Figure S7. Schematic illustration on how to determine the electronic stricture of a semiconductor using valence band, work function and bang gap measurements. To convert the absolute energy scale *vs* vacuum to the redox potential scale *vs* SHE, a scale shift of 4.44 is required, as 4.44 eV on the former corresponds to 0.00 V on the latter, at 298 K. Reproduced from ref [8].

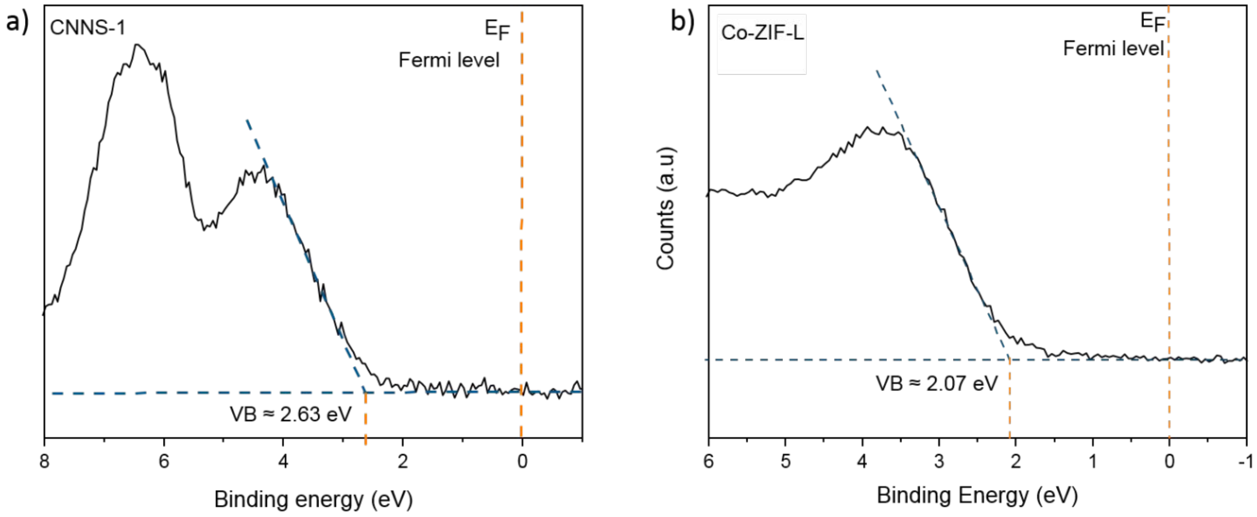

Figure S8. Valence band measurements of a) CNNS-1 and b) Co-ZIF-L.

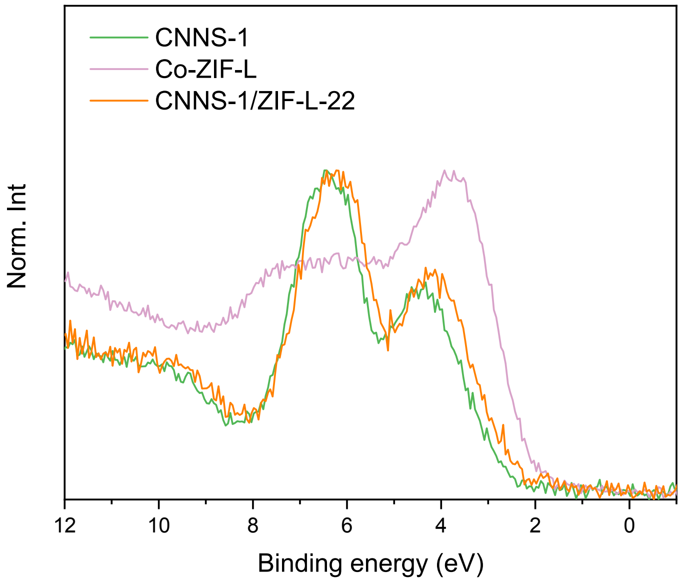

Figure S9. Valence band measurements of CNNS-1, Co-ZIF-L and CNNS-1/ZIF-L-22.

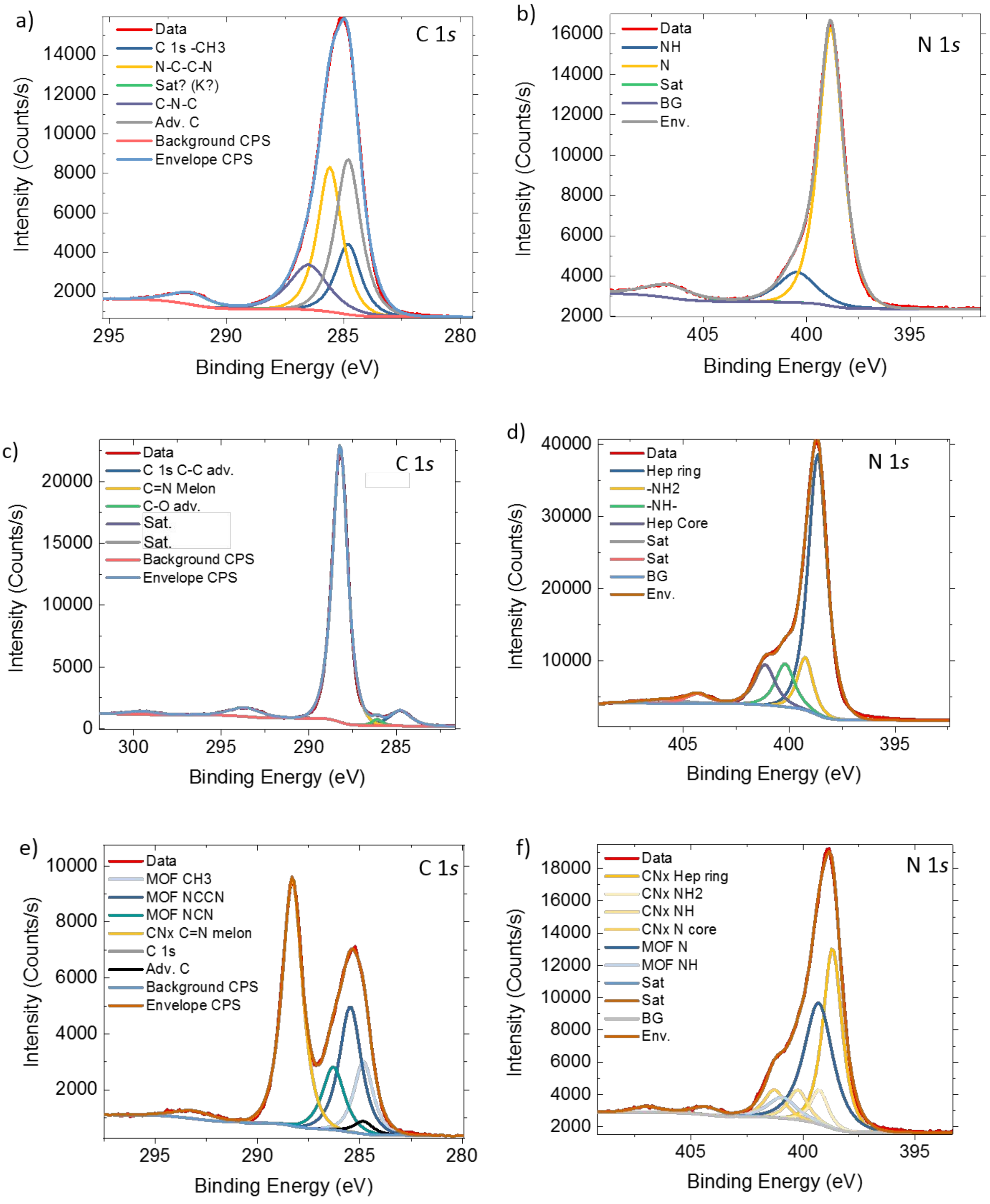

Figure S10. High resolution XPS and peak fittings of the C1*s* and N1*s* core levels in: a), b) Co-ZIF-L; c), d) CNNS-1 and e), f) CNNS-1/ZIF-L-22.

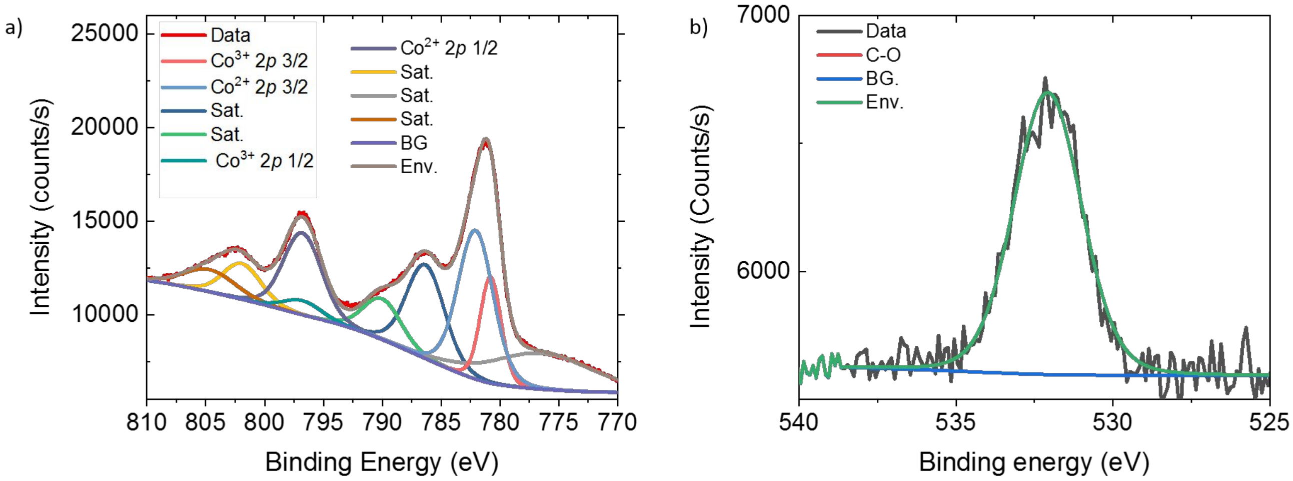

Figure S11. High resolution XPS and peak fittings of Co-ZIF-L in the a) Co *2p* and b) O 1*s* core level regions.

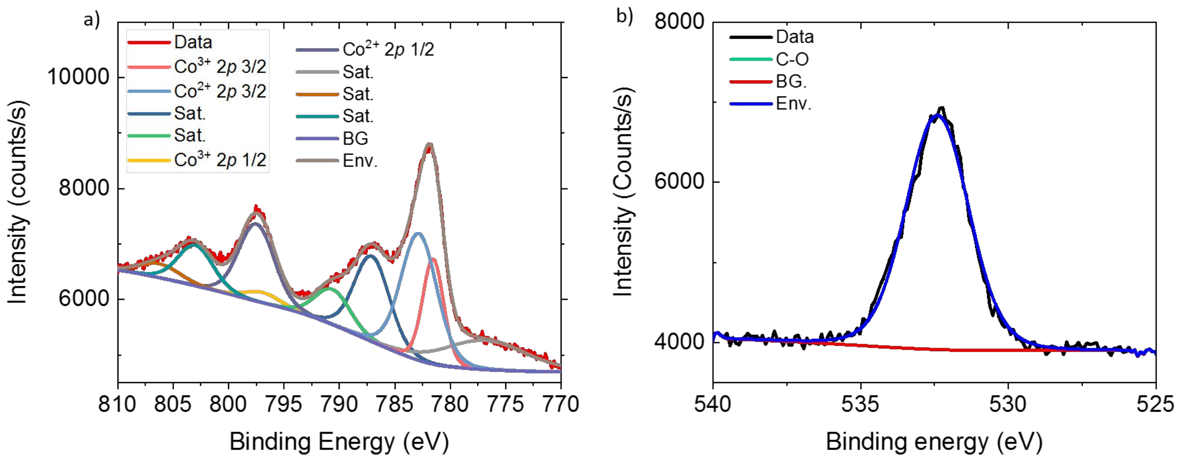

Figure S12. High resolution XPS and peak fittings of CNNS-1/ZIF-L-22 in the Co *2p* and O 1*s* core level regions.

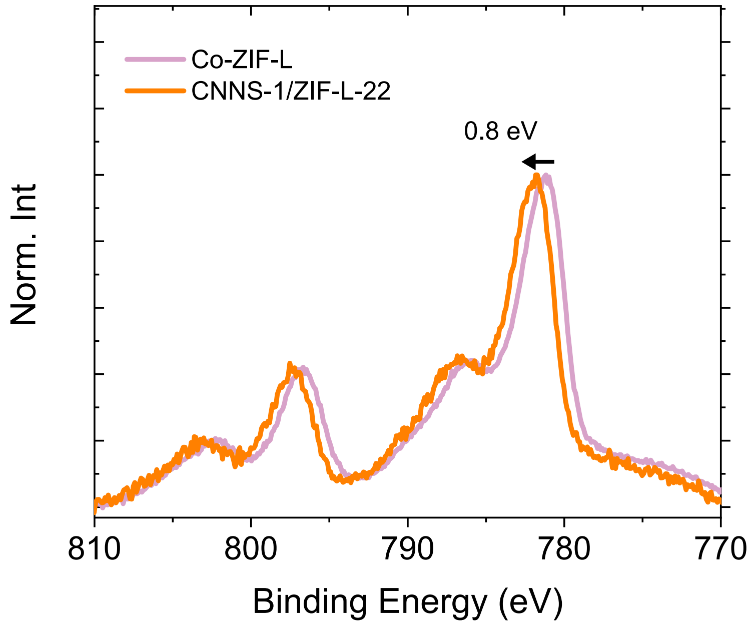

Figure S13. High resolution XPS of Co-ZIF-L and CNNS-1/ZIF-L-22 in the Co 2*p* core level region illustrating the Fermi level shift.

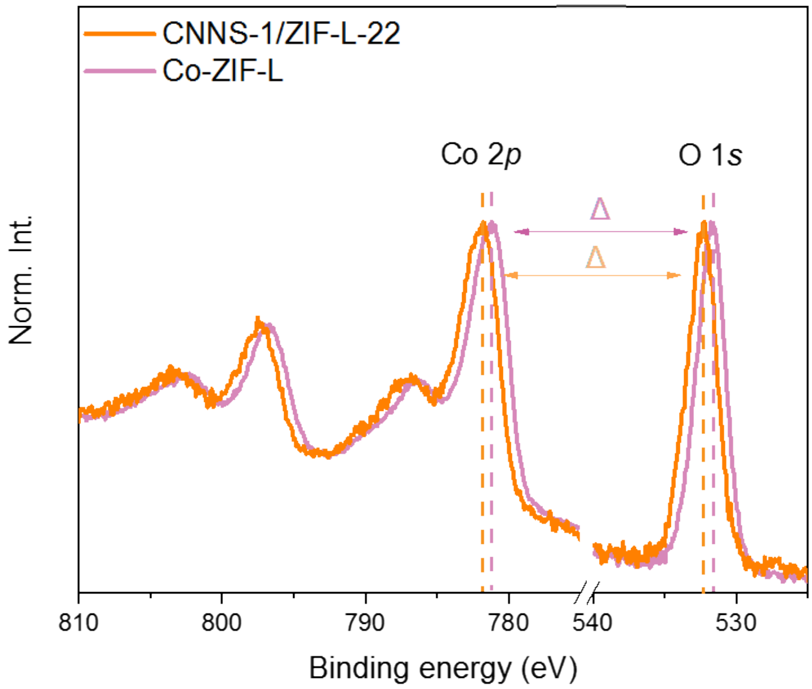

Figure S14. High resolution XPS of Co-ZIF-L and CNNS-1/ZIF-L-22 Co 2*p* and O 1*s* core level spectra showing an identical shift towards lower binding energies upon heterojunction formation. Consequently, the Co 2*p* – O 1*s* peak separation (Δ) is invariant upon heterojunction formation. This observation can be explained by the Equations below (S1-S2).[9, 10] For a given material, referencing the binding energy of one core line against another produces a property that is not sensitive to the position of the Fermi level.

$$\Delta E_B = E_B^o - E_B^i = \left(E_f - E_{core}^o\right) - \left(E_f - E_{core}^i\right) \qquad \text{(S1)}$$

$$\Delta E_B = E_{core}^i - E_{core}^o \qquad \text{(S2)}$$

$E_B^i =$ binding energy of a peak of interest (here O1s); $E_B^o =$ binding energy of the second peak of interest (Co 2*p*); E_{core}^i and E_{core}^o correspond to the absolute energy of these states.

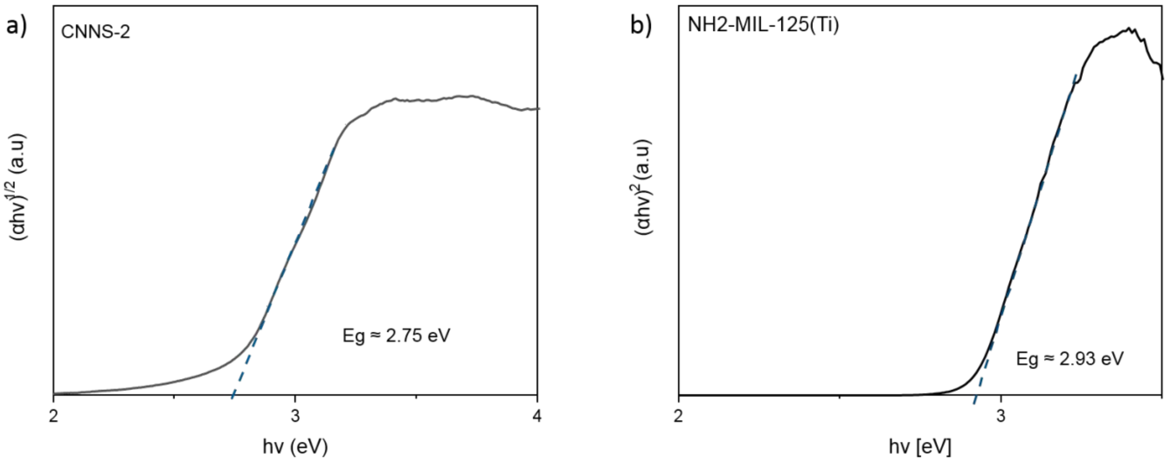

Figure S15. Tauc plots of a) CNNS-2 and b) MIL-125-NH_2.

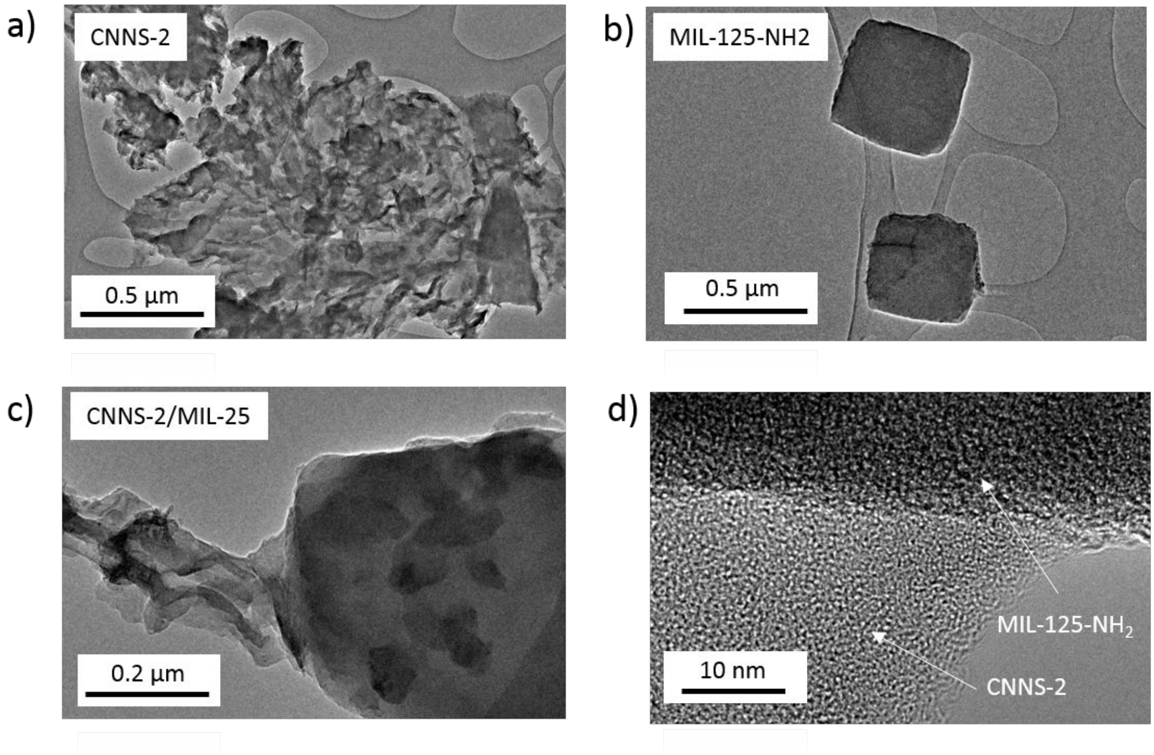

Figure S16. TEM images of: a) CNNS-2, b) MIL-125-NH_2 and c), d) CNNS-2/MIL-25 (High resolution).

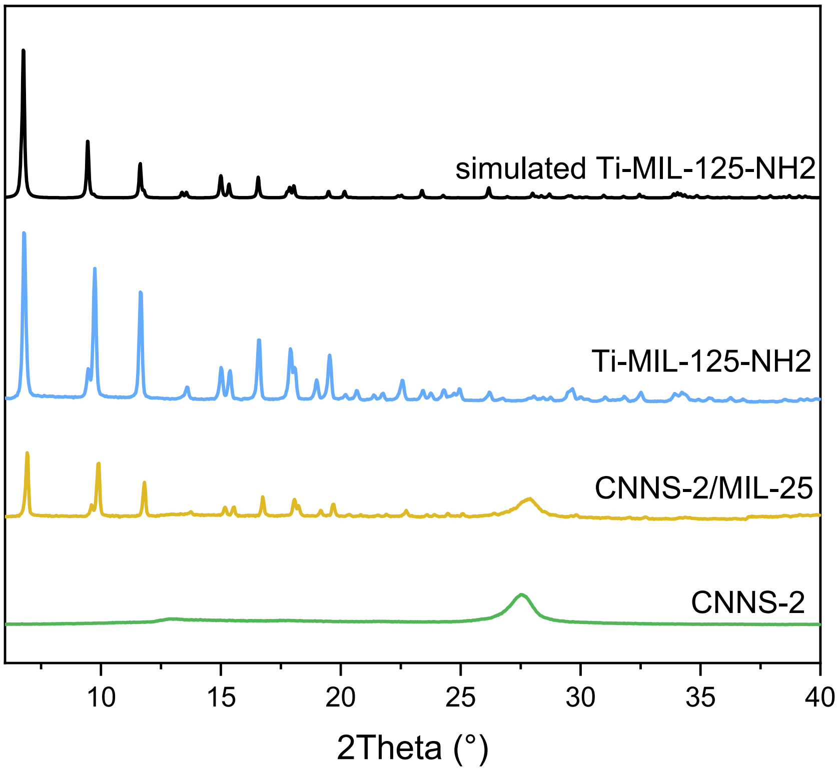

Figure S17. XRD patterns of CNNS-2, MIL-125-NH_2, CNNS-2/MIL-25 and the simulated Ti-MIL-125-NH_2. The measured XRD patterns of Ti-MIL-125-NH_2 are similar to the simulated one, confirming the successful synthesis of Ti-MIL-125-NH_2. In addition, CNNS-2/MIL-25 exhibits a combination of CNNS-2 and Ti-MIL-125-NH_2 patterns, indicating that both CNNS-2 and Ti-MIL-125-NH_2 have been successful incorporate din the heterojunction.

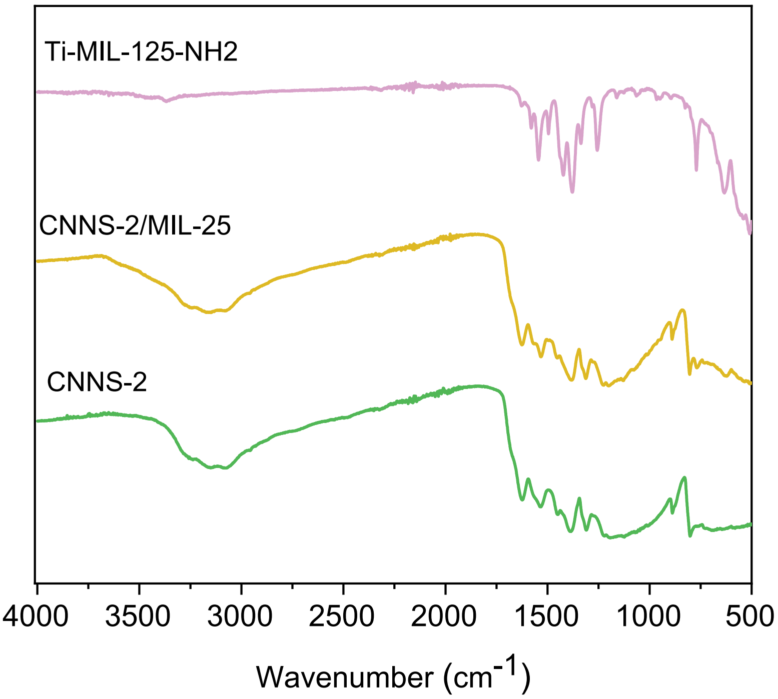

Figure S18. FTIR spectra of CNNS-2, MIL-125-NH_2 and CNNS-2/MIL-25.

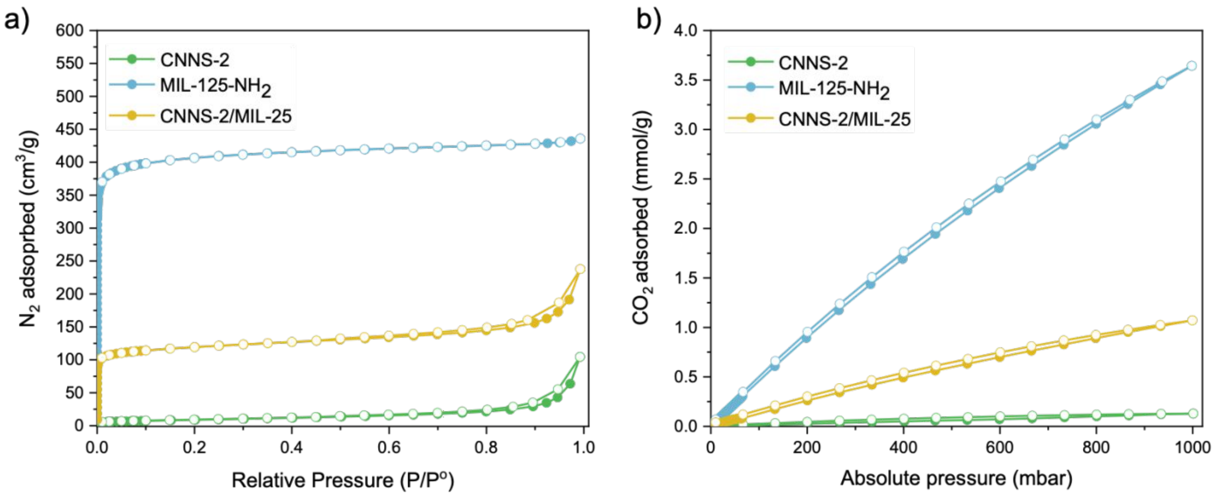

Figure S19. a) N_2 sorption isotherms at 77 K and b) CO_2 sorption isotherms at 298 K.

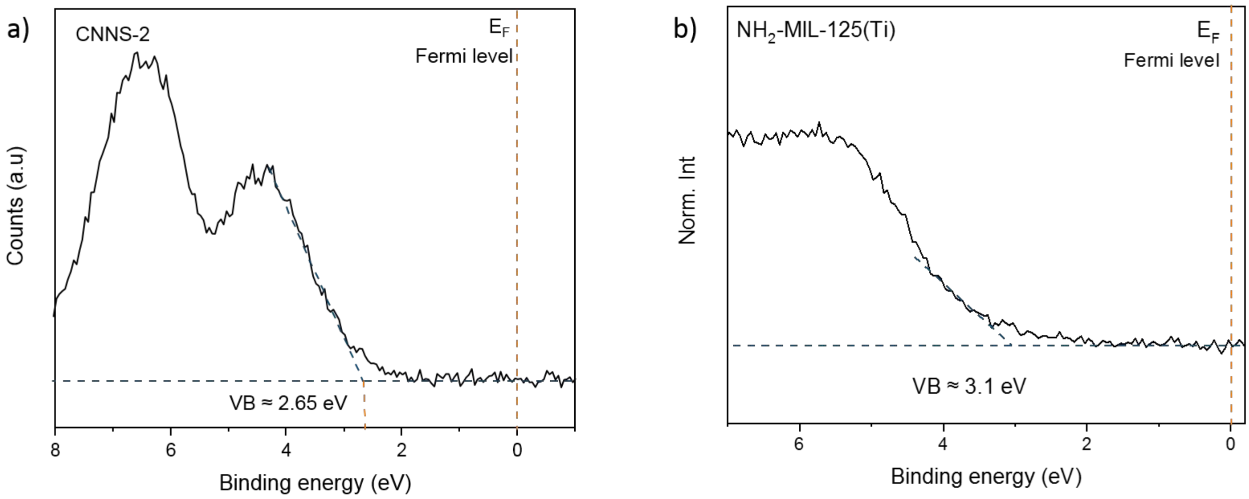

Figure S20. Valence band measurements of a) CNNS-2 and b) MIL-125-NH_2.

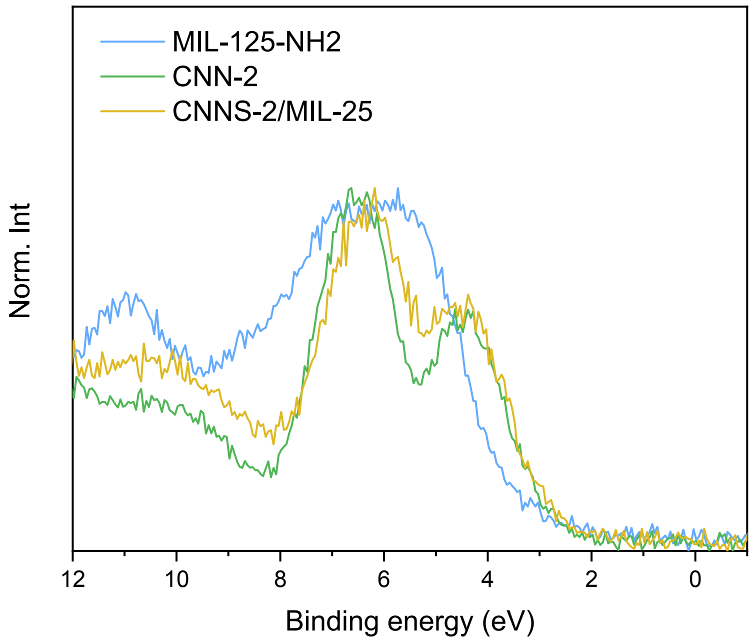

Figure S21. Valence band spectra of CNNS-2, MIL-125-NH_2 and CNNS-2/MIL-25.

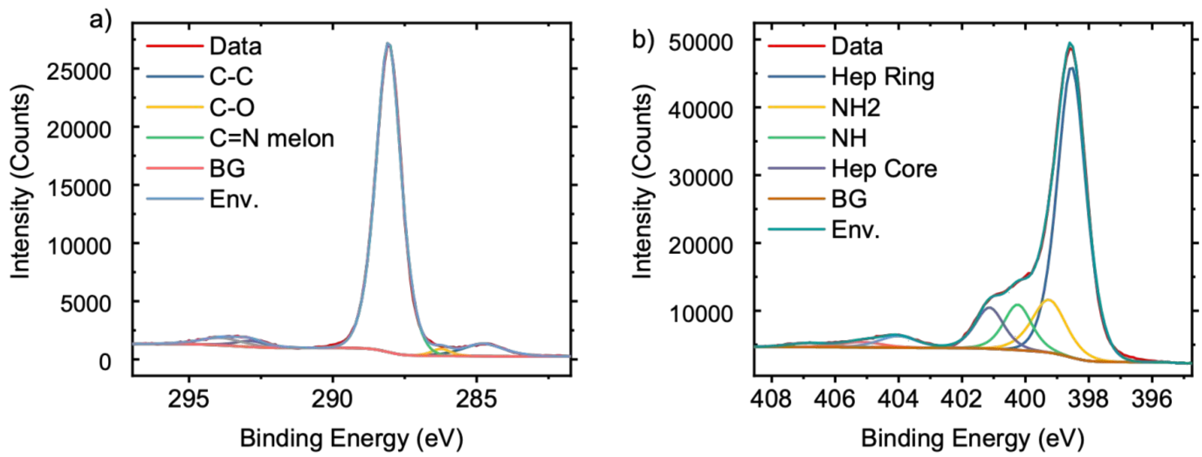

Figure S22. High resolution XPS and peak fittings of CNNS-2 in the a) C1*s* and b) N1*s* core level regions.

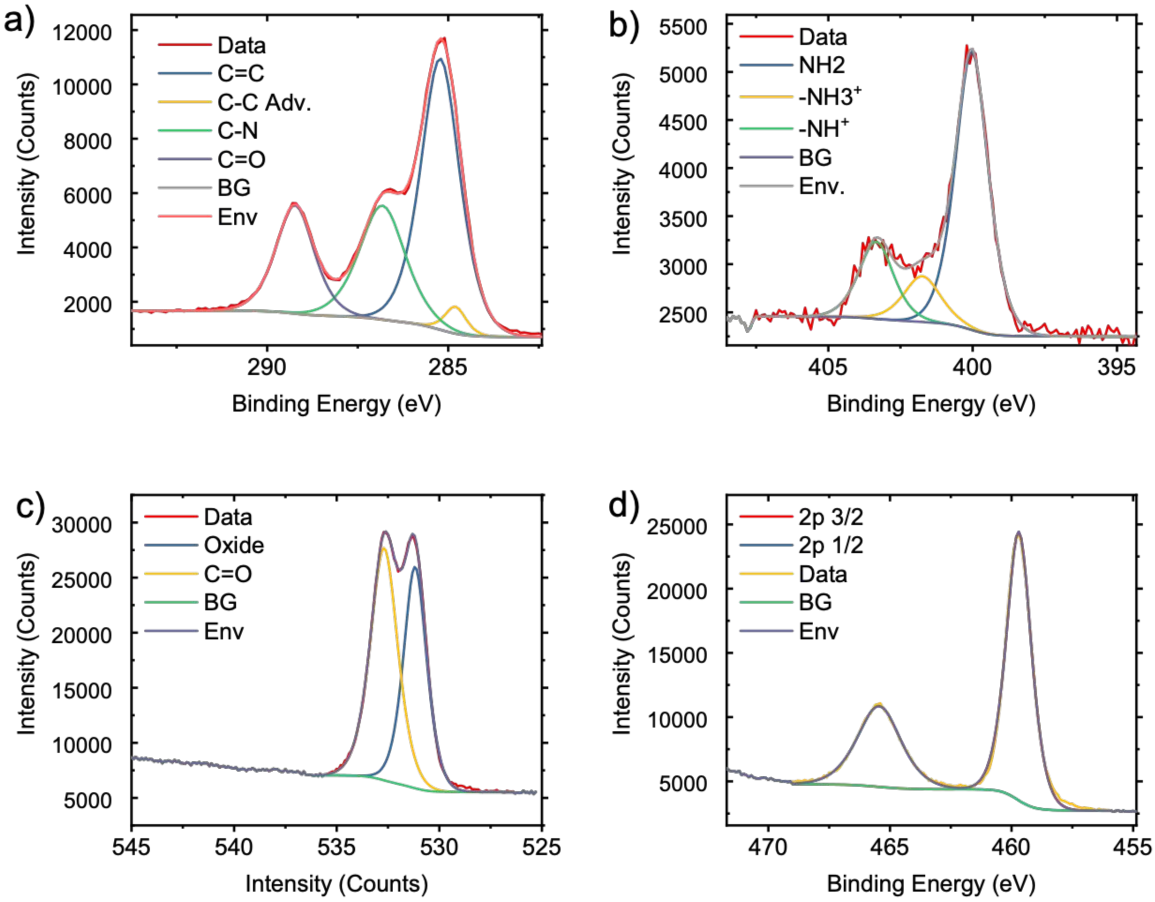

Figure S23. High resolution XPS and peak fittings of MIL-125-NH_2 in the: a) C 1*s*, b) N 1s, c) O 1*s* and d) Ti 2*p* core level regions.

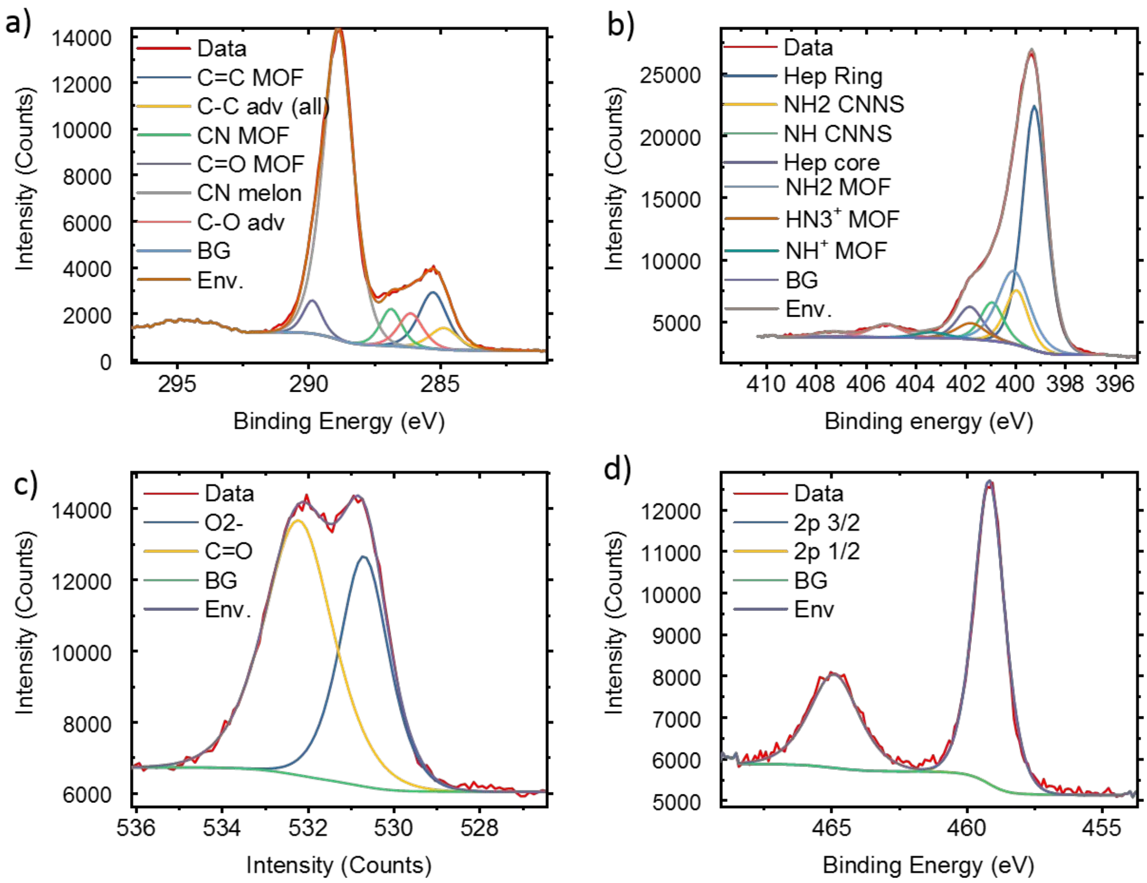

Figure S24. High resolution XPS and peak fittings of CNNS-2/MIL-25 in the: a) C 1*s*, b) N 1*s*, c) O 1*s* and d) Ti 2*p* core level regions.

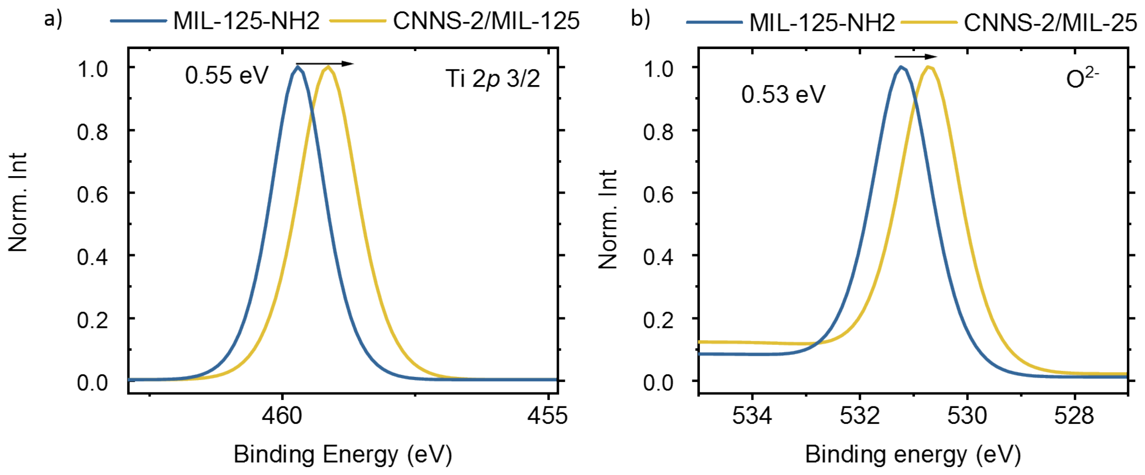

Figure S25. Comparison of MIL-125-NH_2 and CNNS-2/MIL-25 of the XPS in the: a) Ti $2p_{3/2}$ (Ti^{4+}) and b) O 1*s* (metal oxide; O^{2-}) deconvolution, illustrating the Fermi level shift.

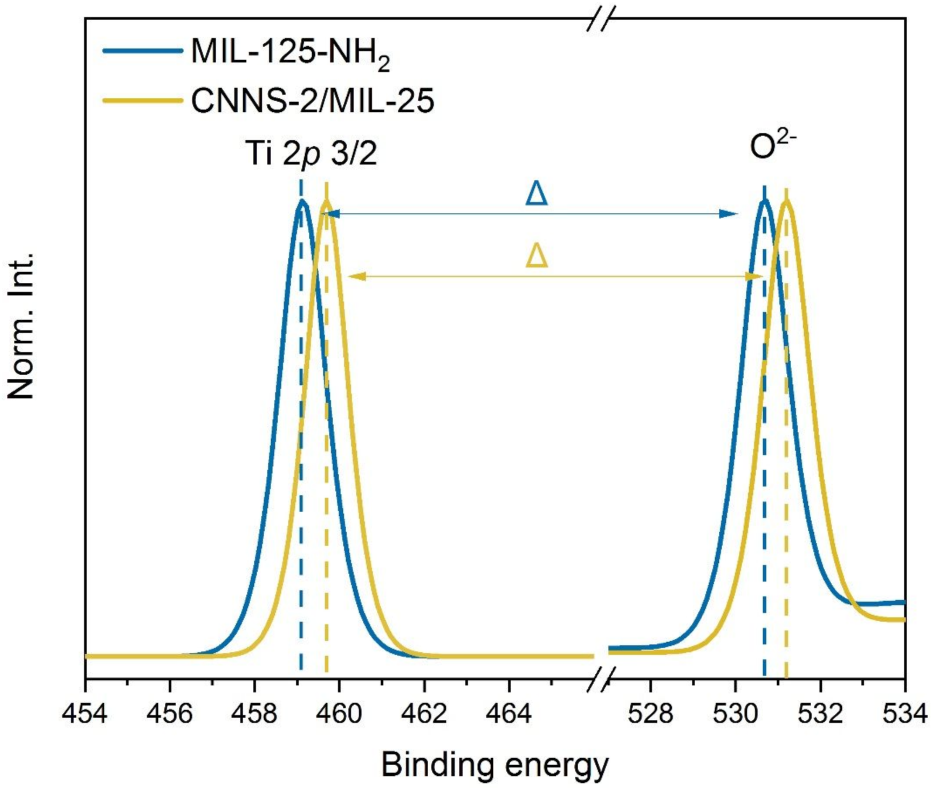

Figure S26. MIL-125-NH_2 and CNNS-2/MIL-25 O^{2-} (oxide) and Ti 2*p* 3/2 components of the O 1*s* and Ti 2*p* spectrum showing an identical shift towards lower binding energies upon heterojunction formation. Consequently, the O^{2-}– Ti 2*p* 3/2 peak separation (Δ) is invariant upon heterojunction formation. This observation can be explained by the Equations below (S3-S4). For a given material, referencing the binding energy of one core line against another produces a property that is not sensitive to the position of the Fermi level.[9, 10]

$$\Delta E_B = E_B^o - E_B^i = \left(E_f - E_{core}^o\right) - \left(E_f - E_{core}^i\right) \qquad \text{(S3)}$$

$$\Delta E_B = E_{core}^i - E_{core}^o \qquad \text{(S4)}$$

$E_B^i =$ binding energy of a peak of interest (here O^{2-}); $E_B^o =$ binding energy of the second peak of interest (Ti 2*p* 2/3); E_{core}^i and E_{core}^o correspond to the absolute energy of these states.

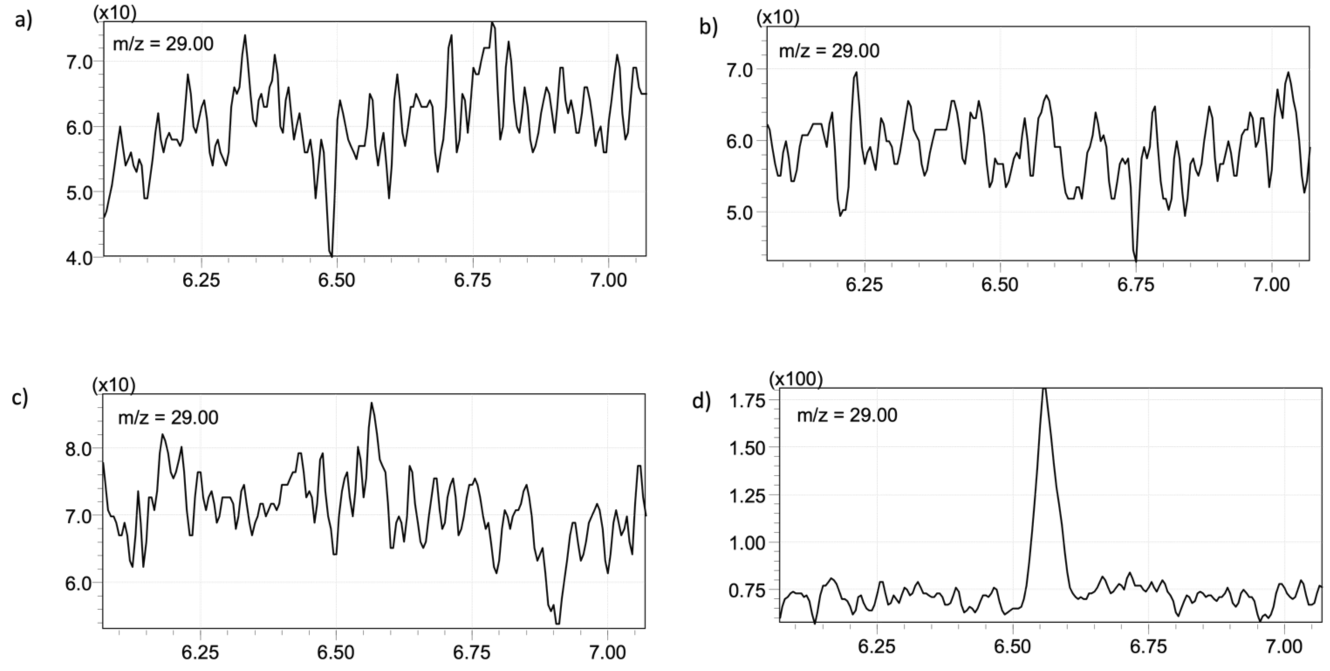

Figure S27. Mass spectrum chromatogram of CNNS-2/MIL-25 illustrating the ^{13}CO (m/z = 29) peak observed with $^{12}CO_2$ after a) 0 min and b) 5 h UV-vis irradiation, and the ^{13}CO (m/z = 29) peak observed with $^{13}CO_2$ after c) 0 min and d) 5 h UV-vis irradiation.

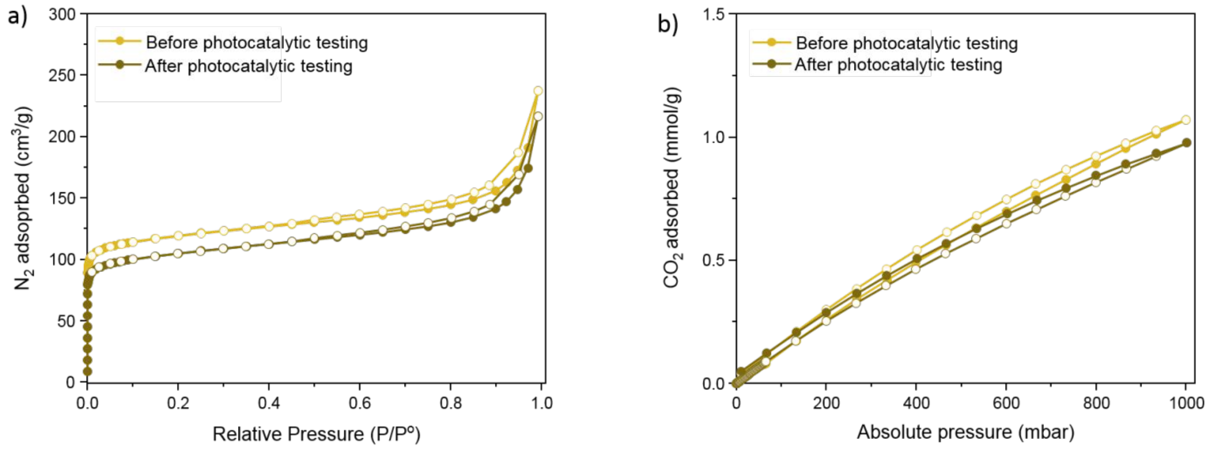

Figure S28. a) N_2 adsorption isotherms at 77 K and b) CO_2 adsorption isotherms at 298 K of CNNS-2/MIL-25 before and after CO_2 photoreduction test.

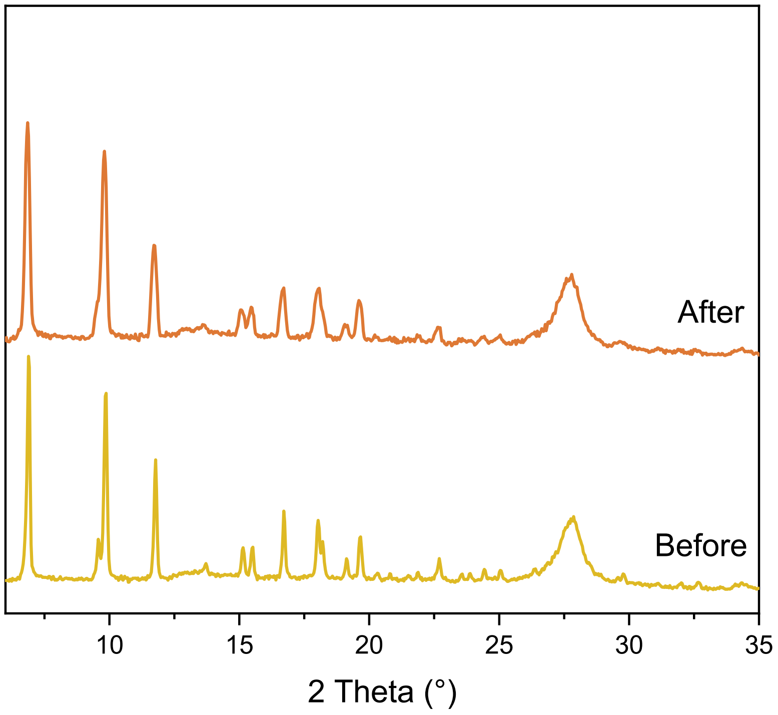

Figure S29. XRD patterns of CNNS-2/MIL-25 before and after CO_2 photoreduction tests.

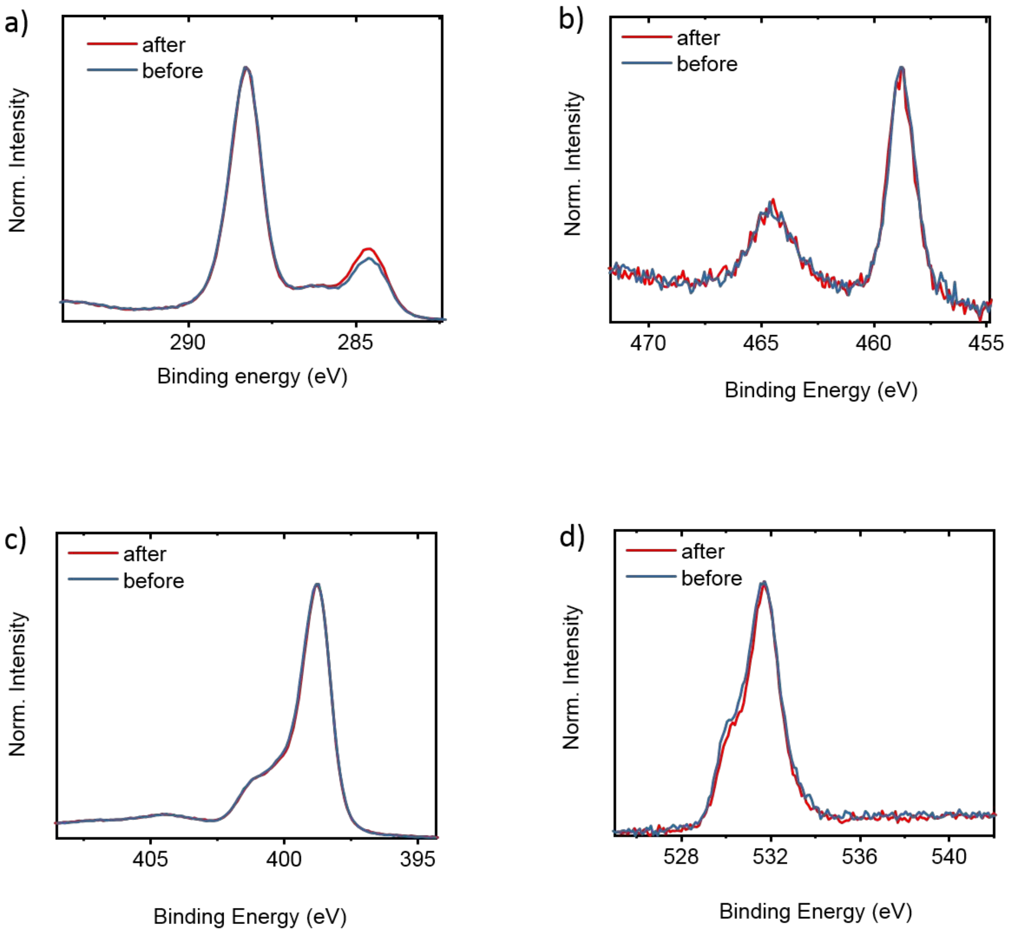

Figure S30. High resolution XPS of CNNS-2/MIL-25 before and after photocatalytic testing in the: a) C 1*s*, b) Ti 2*p* c) O 1*s* and d) N 1*s* core level regions.

2. References

1. Xu, G. L.; Zhang, H. B.; Wei, J.; Zhang, H. X.; Wu, X.; Li, Y.; Li, C. S.; Zhang, J.; Ye, J. H., Integrating the g-C3N4 Nanosheet with B-H Bonding Decorated Metal-Organic Framework for CO2 Activation and Photoreduction. *Acs Nano* **2018,** *12* (6), 5333-5340.
2. Li, X.; Song, X. H.; Ma, C. C.; Cheng, Y. L.; Shen, D.; Zhang, S. M.; Liu, W. K.; Huo, P. W.; Wang, H. Q., Direct Z-Scheme WO3/Graphitic Carbon Nitride Nanocomposites for the Photoreduction of CO2. *Acs Appl Nano Mater* **2020,** *3* (2), 1298-1306.
3. Crake, A.; Christoforidis, K. C.; Godin, R.; Moss, B.; Kafizas, A.; Zafeiratos, S.; Durrant, J. R.; Petit, C., Titanium dioxide/carbon nitride nanosheet nanocomposites for gas phase CO2 photoreduction under UV-visible irradiation. *Appl Catal B-Environ* **2019,** *242*, 369-378.
4. Zhao, Y.; Cai, W.; Chen, J.; Miao, Y.; Bu, Y., A Highly Efficient Composite Catalyst Constructed From NH2-MIL-125(Ti) and Reduced Graphene Oxide for CO2 Photoreduction. *Front Chem* **2019,** *7*, 789.
5. Cheng, X. M.; Gu, Y. M.; Zhang, X. Y.; Dao, X. Y.; Wang, S. Q.; Ma, J.; Zhao, J.; Sun, W. Y., Crystallographic facet heterojunction of MIL-125-NH2(Ti) for carbon dioxide photoreduction. *Appl Catal B-Environ* **2021,** *298*.
6. Wang, L.; Zhang, Z.; Han, Q.; Liu, Y.; Zhong, J.; Chen, J.; Huang, J.; She, H.; Wang, Q., Preparation of CdS-P25/ZIF-67 composite material and its photocatalytic CO2 reduction performance. *Appl Surf Sci* **2022,** *584*, 152645.
7. Shao, W.; Chen, Y. R.; Xie, F.; Zhang, H.; Wang, H. T.; Chang, N., Facile construction of a ZIF-67/AgCl/Ag heterojunction via chemical etching and surface ion exchange strategy for enhanced visible light driven photocatalysis. *Rsc Adv* **2020,** *10* (63), 38174-38183.
8. Shankar R, H. A., Kerherve G, Petit C, Band Positioning in Boron Nitride and Metal-Free Photocatalysts via Photoelectron- and UV-Vis Diffuse Reflectance Spectroscopy. *ChemRxiv. Cambridge: Cambridge Open Engage* **2020**.
9. Moss, B.; Wang, Q.; Butler, K. T.; Grau-Crespo, R.; Selim, S.; Regoutz, A.; Hisatomi, T.; Godin, R.; Payne, D. J.; Kafizas, A.; Domen, K.; Steier, L.; Durrant, J. R., Linking in situ charge accumulation to electronic structure in doped SrTiO3 reveals design principles for hydrogen-evolving photocatalysts. *Nat Mater* **2021**.
10. Glover, E. N. K.; Ellington, S. G.; Sankar, G.; Palgrave, R. G., The nature and effects of rhodium and antimony dopants on the electronic structure of TiO2: towards design of Z-scheme photocatalysts. *J Mater Chem A* **2016,** *4* (18), 6946-6954.
